# Clonotype-enriched somatic hypermutations drive affinity maturation of a public human antibody targeting an occluded sarbecovirus epitope

**DOI:** 10.1016/j.celrep.2025.116122

**Published:** 2025-08-12

**Authors:** Vishal N. Rao, Iden A. Sapse, Hallie Cohn, Duck-Kyun Yoo, Pei Tong, Jordan J. Clark, Bailey Bozarth, Yuexing Chen, Komal Srivastava, Gagandeep Singh, Florian Krammer, Viviana Simon, Duane R. Wesemann, Goran Bajic, Camila H. Coelho

**Affiliations:** 1 Department of Microbiology, Icahn School of Medicine at Mount Sinai, New York, NY, USA; 2 Center for Vaccine Research and Pandemic Preparedness, Icahn School of Medicine at Mount Sinai, New York, NY, USA; 3 Graduate School of Biomedical Sciences, Icahn School of Medicine at Mount Sinai, New York, NY, USA; 4 Department of Medicine, Division of Allergy and Clinical Immunology, Division of Genetics, Brigham and Women’s Hospital, Boston, MA, USA; 5 Department of Pathology, Molecular and Cell-Based Medicine, Icahn School of Medicine at Mount Sinai, New York, NY, USA; 6 Ignaz Semmelweis Institute, Interuniversity Institute for Infection Research, Medical University of Vienna, Vienna, Austria; 7 The Global Health and Emerging Pathogens Institute, Icahn School of Medicine at Mount Sinai, New York, NY, USA; 8 Division of Infectious Diseases, Department of Medicine, Icahn School of Medicine at Mount Sinai, New York, NY, USA; 9 Harvard Medical School, Boston, MA, USA; 10 The Broad Institute of MIT and Harvard, Cambridge, MA, USA; 11 The Ragon Institute of MGH, MIT, and Harvard, Cambridge, MA, USA; 12 Massachusetts Consortium on Pathogen Readiness, Boston, MA, USA; 13 Precision Immunology Institute, Icahn School of Medicine at Mount Sinai, New York, NY, USA; 14 Lead contact

## Abstract

Investigating public antibodies that recognize conserved epitopes is critical for vaccine development. Identifying somatic hypermutations (SHMs) that enhance antigen affinity in these public antibodies is key to guiding vaccine design for better protection against pathogens. We propose that affinity-enhancing SHMs are selectively enriched in public antibody clonotypes, surpassing the background frequency seen in antibodies carrying the same V genes but with different epitope specificities. Using M15, a human *IGHV4-59*/*IGKV3-20* public antibody as a model, we compare SHM signatures in antibodies that use the same V genes but recognize other epitopes. We identified clonotype-enriched mutations in the light chain of M15 and showed that, in combination, these SHMs enhance binding to a previously uncharacterized Sarbecovirus epitope, with antibody responses to it increasing after sequential vaccination. Our findings identify convergence and clonotype enrichment as features of affinity-enhancing SHMs in public antibodies, which can help guide vaccine design aimed at eliciting such antibodies.

## In brief

Rao et al. demonstrate that convergent somatic hypermutations in the light chain, enriched within a sarbecovirus-specific public antibody clonotype, drive affinity maturation. This antibody targets a previously undescribed central interface epitope, occluded in pre-fusion spike, with repeated SARS-CoV-2 vaccinations boosting serum antibodies targeting this epitopic region.

## Graphical abstract

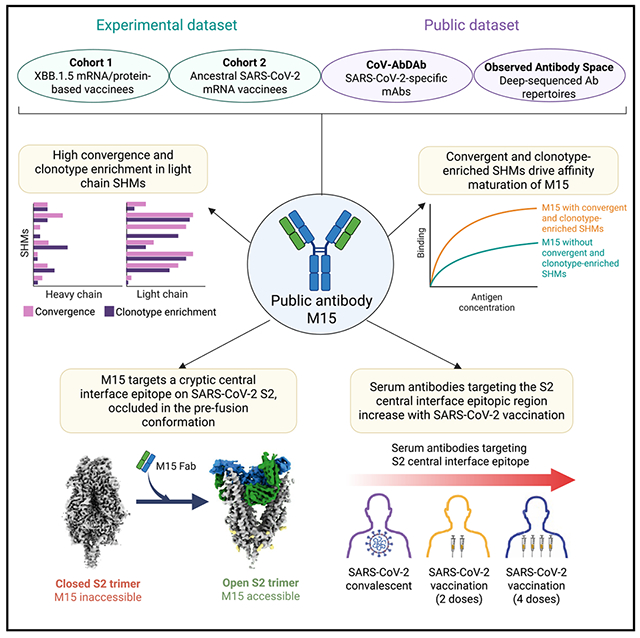

## INTRODUCTION

The adaptive humoral immune system recognizes a wide range of antigens due to the vast diversity of the antibody repertoire. Stochastic recombination of germline variable (V), diversity (D), and joining (J) antibody genes, coupled with diversity in the junctional regions, gives rise to over 10^11^ possible combinations, referred to as clonotypes.^1,2^ Despite this extensive diversity, different individuals can generate the same clonotypes in response to a given antigen, known as public antibodies. Public antibodies have been identified in response to various pathogens, including severe acute respiratory syndrome coronavirus 2 (SARS-CoV-2), human immunodeficiency virus 1 (HIV-1), influenza virus, dengue virus, Zika virus, and Ebola virus.^3–9^ The recurring presence of a public antibody suggests that certain germline gene segments possess paratope features that improve binding to the antigenic target.^10^ In addition to V(D)J recombination, another key feature that contributes to the diversity of antibodies is somatic hypermutation (SHM), which can improve affinity for a specific antigen.^11–14^ Previous studies have shown that key SHMs acquired during affinity maturation of public antibodies are convergently selected across different individuals.^3,15^ Thus, public antibodies serve as a valuable model to investigate how SHM shapes antibody evolution in humans.

Affinity maturation occurs in germinal centers within secondary lymphoid organs where B cells acquire SHMs in the dark zone and then shuttle to the light zone, where selection based on affinity takes place.^12,16^ This spatial separation suggests that SHMs in the dark zone are initially acquired independently of their potential to enhance affinity. As a result, SHMs can be neutral, disruptive, or beneficial for affinity maturation, and only a subset of these mutations is retained during clonal expansion.^17,18^ Identifying affinity-enhancing SHMs in the backdrop of other SHMs remains a challenging question.

While affinity-enhancing SHMs are known to be convergently selected across individuals,^3,15^ not all convergent SHMs are necessarily affinity enhancing. Some may occur frequently due to the selective targeting of the activation-induced cytidine deaminase enzyme responsible for SHM at certain hotspot motifs.^19,20^ If these convergent SHMs do not selectively contribute to the affinity maturation of a particular clonotype, they would likely appear at a background frequency in B cells, regardless of epitope specificity. The latter assumption is further supported by the fact that antibodies recognizing different epitopes often utilize the same antibody V gene, which contributes to major interactions with epitopes.^21^

In this context, we hypothesize that affinity-enhancing SHMs in public antibody lineages are not only convergent but also “clonotype enriched,” which we define here as SHMs enriched in the public clonotype compared with other clonotypes that use the same V gene but target different epitopes. To investigate this hypothesis, we focus on M15, a newly discovered public antibody targeting the S2 domain of the SARS-CoV-2 spike protein. We show that the affinity maturation of M15-like antibodies is driven by convergent and clonotype-enriched SHMs, which enhance binding to a previously uncharacterized central interface (CI) epitope exposed in the open conformation of S2. We also show that serum antibodies targeting this epitopic region increase in proportion upon multiple vaccinations. Overall, our analysis provides a framework for identifying affinity-enhancing SHMs, which can be leveraged to optimize the design of vaccine immunogens that induce favorable affinity maturation pathways.

## RESULTS

### M15 is a human public antibody elicited specifically upon exposure to SARS-CoV-2 in multiple cohorts

Paired heavy and light chain cDNA sequences of 603 single-cell plasmablasts from five individuals who received either the XBB.1.5 monovalent mRNA booster (BioNTech/Pfizer or Moderna) or the protein-based booster (Novavax) in 2023 (Cohort 1)^22^ were analyzed to identify public antibodies binding to the spike protein of SARS-CoV-2 (Table S1). To identify the most public antibody in Cohort 1, we mapped each sequence from our repertoire to a database of SARS-CoV-2-specific antibody sequences (database: CoV-AbDab),^23^ based on identical V and J gene usage and greater than 70% CDR3 amino acid similarity in both heavy and light chains from the repertoire, as this would ensure that the identified public antibodies from different individuals would have evolved from highly similar germline B cells.^24,25^ Based on these criteria, four sequences from Cohort 1 (antibodies M13, M14, M15, and M30) mapped to at least one antibody in the database, whereas none of the antibodies from the Novavax vaccinee cohort (protein-based vaccine) were mapped to sequences deposited in CoV-AbDab (Table S2). One of the antibodies, M15, was found to map to 17 distinct antibodies reported across seven studies of SARS-CoV-2-infected or vaccinated individuals, suggesting that it may be a widely elicited public antibody.

Next, to validate M15 as a public antibody, we screened single-cell V(D)J sequences from plasmablasts and spike-specific memory B cells in a cohort of SARS-CoV-2 mRNA vaccinees (cohort 2, *n* = 19; Table S1) and two additional datasets: (1) database: Observed Antibody Space (OAS), comprising antibody repertoires elicited under different conditions, including tonsillitis, obstructive sleep apnea, HIV-1 infection, and SARS-CoV-2 infection,^26^ and (2) a dataset that we compiled in-house, containing 3,734 sequences of monoclonal antibodies (mAbs) elicited upon influenza virus infection or vaccination from 24 studies and *Plasmodium falciparum* (malaria) vaccination from two studies (Table S3). These sequences were also mapped to M15 based on identical V and J germline gene usage, along with a CDR3 amino acid similarity of 70% or greater in both the heavy and light chains. Only individuals exposed to SARS-CoV-2 through infection or vaccination exhibited M15-like sequences in their repertoire, further confirming the antigen specificity of M15 to SARS-CoV-2 (Table 1). Among four studies involving individuals infected with SARS-CoV-2, all participants in two of the studies^27,28^ harbored M15-like sequences (Figure S1A), contributing a total of 122 sequences similar to M15. By combining all M15-like sequences from cohort 1, cohort 2, and the CoV-AbDab and OAS databases, we identified a total of 147 M15-like sequences. These sequences were found across various B cell subtypes, including plasmablasts, memory B cells, and naive B cells in peripheral blood, as well as germinal center B cells and plasma cells in draining lymph nodes (Figure S1B).

### M15 binds broadly to the S2 domain of the spike protein of sarbecoviruses

**Figure 1.**
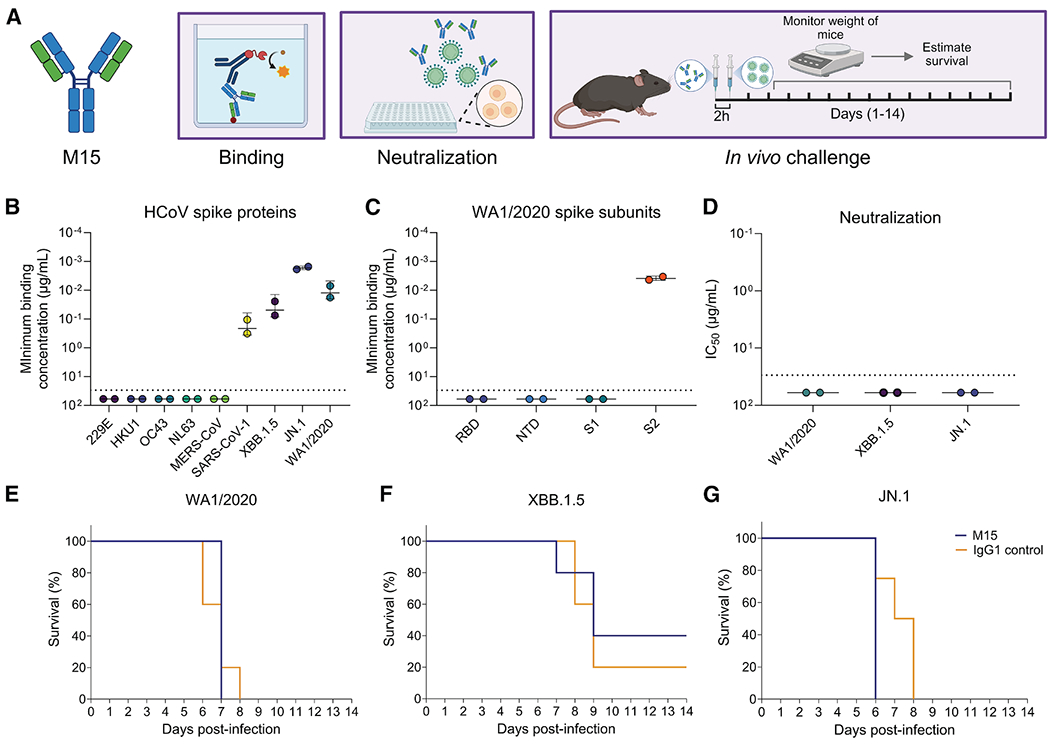
Binding, neutralization, and *in vivo* protection activity of M15 (A) Schematic of binding, *in vitro* neutralization, and *in vivo* protection experiments performed using M15, created with BioRender. (B and C) Binding activity of M15 to (B) spike proteins from human coronaviruses, including the XBB.1.5 and JN.1 variants of SARS-CoV-2, and (C) subunits of the WA1/2020 (ancestral strain) spike protein, represented as the minimum binding concentration (μg/mL) measured by ELISA. The dotted line represents the limit of detection (LOD), set at the starting dilution of 30 μg/mL. All values with a minimum binding concentration of >30 μg/mL were set to 60 μg/mL for graphing purposes. Data are represented as mean ± SD. (D) *In vitro* neutralization capacity of M15 against WA1/2020, XBB.1.5, and JN.1 variants, represented as half-maximal inhibitory concentrations (IC_50_). All values with an IC_50_ of >30 μg/mL were set to 60 μg/mL for graphing purposes. Data are represented as mean ± SD. (E–G) Survival curves of hACE2-K18 mice prophylactically treated with 10 mg/kg (intraperitoneal) of M15 antibody before challenge with a 3×LD_50_ dose of (E) WA1/2020, (F) XBB.1.5, or (G) JN.1. CR9114, an isotype-matched influenza virus anti-hemagglutinin mAb, was used as a negative control mAb.

We evaluated the binding of M15 to SARS-CoV-2 spike proteins of WA1/2020 (ancestral strain), XBB.1.5, and JN.1 variants, in addition to human coronaviruses (HCoVs) 229E, HKU1, OC43, NL-63, MERS-CoV, and SARS-CoV (Figure 1A). M15 bound to SARS-CoV-1 and all SARS-CoV-2 variants tested, but not the other HCoV spike proteins, suggesting broad binding across sarbecoviruses (Figure 1B). We then tested the binding of M15 to individual subunits of the SARS-CoV-2 USA-WA1/2020 spike protein and found that it specifically binds to the highly conserved S2 domain (Figure 1C). This finding potentially explains its broad binding activity against sarbecoviruses (Figure 1B). However, M15 did not neutralize WA1/2020, XBB.1.5, or JN.1 variants *in vitro* (Figures 1D and S2) and was unable to protect transgenic mice expressing the human ACE2 receptor (hACE2-K18) against viral challenge (Figures 1E–1G). We also confirmed that other M15-like antibodies from the CoV-AbDab database bind to the S2 subunit of the spike protein of sarbecoviruses and lack neutralization capacity against SARS-CoV-2 (Figure S2). Having characterized the subunit specificity of M15, we addressed our hypothesis that convergent and clonotype-enriched SHMs enhance the affinity of M15 with its cognate antigen, S2.

### Convergent and clonotype-enriched SHMs are exclusively found in the M15 light chain and drive affinity maturation

**Figure 2.**
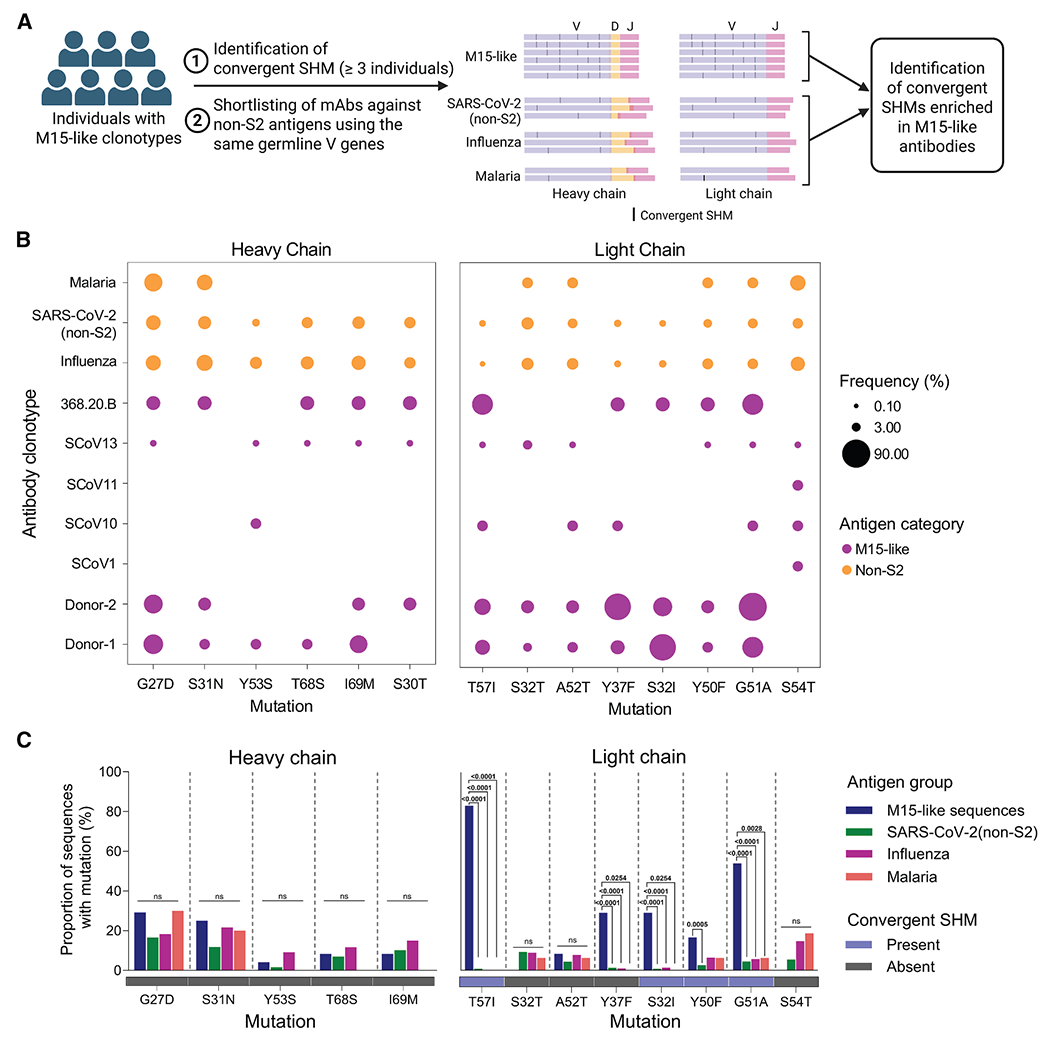
Convergent and specific SHM in M15-like antibodies (A) Schematic workflow for identifying SHMs that are convergent and specific to M15-like antibodies, created with BioRender. Black traces refer to convergent SHMs. The number of sequences illustrated per group is not representative of the actual numbers. Since the non-M15-like sequences (SARS-CoV-2 non-S2, influenza virus, and malaria) share the same germline V gene (*IGH4-59* and *IGKV3-20*) but have different D and J genes, the lengths of the D and J gene segments vary in the cartoon. (B) Frequencies of heavy- (left) and light-chain (right) SHMs in M15-like clonal lineages (126 sequences) and negative-control malaria (heavy chain: 10 sequences, light chain: 16 sequences), influenza virus (heavy chain: 134 sequences, light chain: 441 sequences), and non-S2 (heavy chain: 208 sequences, light chain: 648 sequences) mAbs from CoV-AbDab using the same germline V genes. Mutations present in at least three individuals are shown. (C) The bar plots represent the frequencies of heavy- (left) and light-chain (right) SHM in M15-like singleton sequences from cohort 1, cohort 2, and CoV-AbDab, 21 sequences not previously included in (B), as well as non-S2 antibodies against malaria, influenza virus, and non-S2 epitopes from CoV-AbDab, all using the same germline V genes as above. Underneath the *x* axis, a heatmap illustrates the presence or absence of the convergent SHMs observed in the original M15 sequence. *p* values for pairwise comparisons of frequencies of convergent SHMs between M15-like antibodies and antibodies against malaria, influenza virus, and non-S2 epitopes from CoV-AbDab were obtained using the chi-squared test with Yates’ correction.

We first investigated whether the 147 M15-like sequences identified by matching M15 with cohort 2, CoV-AbDab, and OAS could be leveraged to identify convergent SHMs in this public antibody clonotype. We reconstructed clonal lineages from seven individuals who each presented at least three M15-like sequences, namely, donor 1, donor 2, SCoV-1, SCoV-10, SCoV-11, and SCoV-13 from OAS and one individual, 368.20.B, from CoV-AbDab. To assess convergence, we shortlisted SHMs observed in at least three of the seven individuals and computed the frequency of these mutations within each lineage (Figures 2A and 2B). This yielded five and eight convergent SHMs in the heavy and light chains, respectively. Most of the convergent mutations were contributed by donor 1, donor 2, and 368.20.B, which could be explained by higher overall SHM in these individuals (Figures S3A and S3B).

Next, to assess clonotype enrichment of these convergent SHMs in the heavy and light chains, we compared the frequencies of the convergent mutations with mAbs using the same heavy-(*IGHV4-59*) or light-chain (*IGKV3-20*) V gene as M15, respectively, and specific to non-S2 epitopes from three groups: (1) influenza virus infection/vaccination, (2) malaria-vaccination, and (3) SARS-CoV-2-specific mAbs from CoV-AbDab-recognizing epitopes outside the S2 domain, henceforth referred to as the non-S2 group. These mAbs share the same heavy- or light-chain V gene as M15 but belong to clonotypes with distinct specificities due to variations in junctional regions and heavy-/light-chain pairings.^29^ Notably, the frequencies of convergent heavy-chain mutations showed no significant differences between M15-like clonotypes and mAbs from the non-S2 group. On the other hand, the light-chain mutations S32I, Y37F, Y50F, G51A, and T57I were more frequent in M15-like clonotypes (Figure 2B). We also compared the frequencies of these convergent mutations in the heavy and light chains of M15-like sequences from cohort 1, cohort 2, and CoV-AbDab with the non-S2 group (Figure 2C). The frequency of heavy-chain mutations did not differ between groups, whereas light-chain mutations S32I, Y37F, Y50F, G51A, and T57I were significantly enriched in M15-like antibodies compared with our controls (non-S2 SARS-CoV-2 mAbs, influenza mAbs, or malaria mAbs). Notably, Y50F was only significantly enriched in comparison to SARS-CoV-2 (non-S2) antibodies, suggesting a clonotype-enriched SHM with relatively low convergence compared with the other light-chain SHMs described (Figure 2C). While none of the convergent mutations in the heavy chain appeared in the original sequence of M15, four of the eight convergent mutations in the light chain, namely S32I, Y50F, G51A, and T57I, were present in the original M15 mAb sequence (Figure 2C).

**Figure 3.**
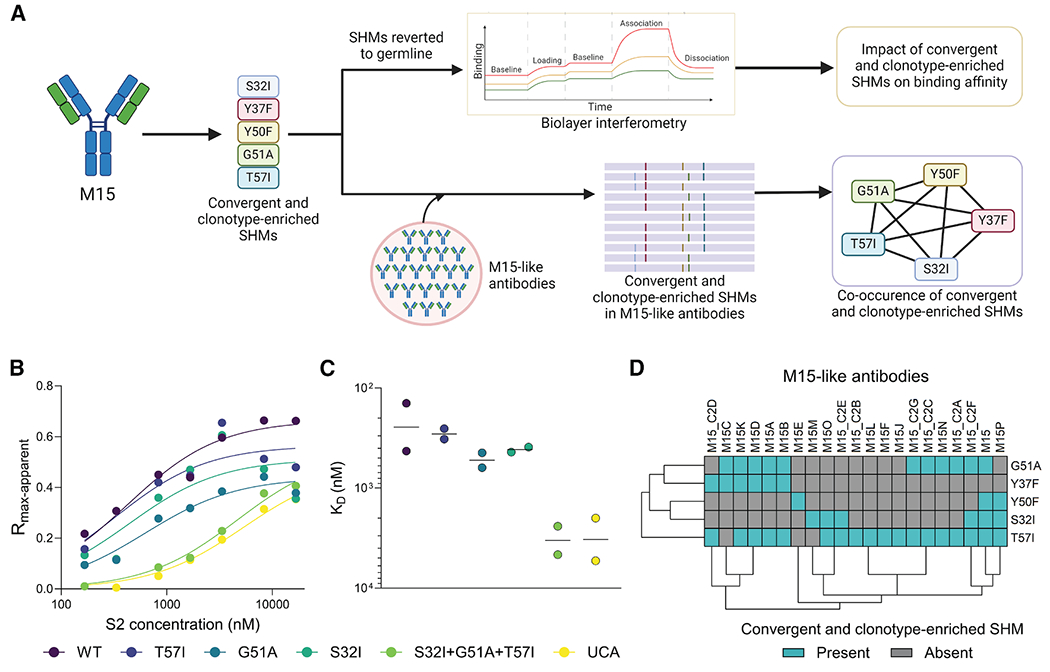
Influence of convergent and clonotype-enriched light-chain SHMs on the binding affinity of M15 (A) Schematic workflow for assessing the role of convergent and clonotype-enriched mutations in affinity maturation of M15 and co-occurrence of these SHMs in M15-like antibodies, created with BioRender. (B) Biolayer interferometry (BLI) is used to assess the binding of M15; single reverted mutants G51A, T57I, and S32I; a triple mutant with all three mutations reverted; and the unmutated common ancestor (UCA) with S2. R_max-apparent_ represents the baseline-corrected maximum binding signal reached during the association step of the assay, measured at different concentrations of S2, ranging from 166.7 to 16,666.7 nM. The lines represent the best-fit one-site specific binding curves. The BLI assay was performed in duplicates for each antibody (only one of the replicates shown). (C) Binding affinity values of M15, the single mutants, the triple mutant, and the UCA, computed as the dissociation constant, K_D_. K_D_ values were obtained from the best-fit one-site specific binding in GraphPad Prism independently for the duplicates. Data are represented as geometric mean of K_D_ values. (D) Biclustered heatmap representing the occurrence of convergent and clonotype-enriched SHMs in M15-like antibodies from cohort 1 (M15), cohort 2 (M15_C2A-F), and CoV-AbDab (M15A-Q). Biclustering was performed using k-means clustering in the “pheatmap” package in R.

Having identified these four mutations in M15, we tested our hypothesis that these convergent and clonotype-enriched SHMs enhance affinity to the antigen S2. We individually reverted the three convergent and clonotype-enriched mutations present in M15, which showed significantly higher frequencies in M15-like antibodies compared with the other three antigen groups (S32I, G51A, and T57I), back to their germline residues. These mutations were also reverted in combination (triple mutant) to assess their impact on binding affinity to the S2 region of wild-type SARS-CoV-2 (Figures 3A–3C). While the single mutants showed a slight decrease in binding affinity (S32I, 1.45-fold; G51A, 1.88-fold; T57I, 1.02-fold) compared with M15 (K_D_ 95% CI, 246.3–709.7 nM), the triple mutant showed a 12-fold decrease, roughly matching the K_D_ of the unmutated common ancestor (K_D_ 95% CI, 3,560–8,522 nM), implying a synergistic effect of mutations S32I, G51A, and T57I in driving affinity maturation of M15 (Figures 3B and 3C).

To explore this further, we examined whether convergent and clonotype-enriched SHMs are acquired simultaneously during affinity maturation. None of the M15-like antibodies from cohort 2 and CoV-AbDab presented all five convergent and clonotype-enriched mutations (Figure 3D). Among the M15-like antibodies, the original M15 antibody from cohort 1 presented the highest number of convergent and clonotype-enriched SHMs (*n* = 4; Figure 3D). We then conducted a linkage analysis to examine the co-occurrence of convergent mutations in both heavy and light chains. Specifically, for each pair of convergent mutations, we created a contingency table to assess their presence within individual lineages. We computed an odds ratio (OR) of co-occurrence for each pair of mutations and considered two mutations to be linked in a lineage if the OR was greater than 2 (Figures S4A–S4F). Among the pairs of convergent mutations, G51A-T57I, which was a pair of convergent and enriched SHMs in M15-like antibodies, exhibited the highest linkage (present in five lineages), followed by G51A-A52T (present in four lineages; Figure S4F). These results suggest that convergent and clonotype-enriched SHMs are critical in the process of affinity maturation of M15.

**Figure 4.**
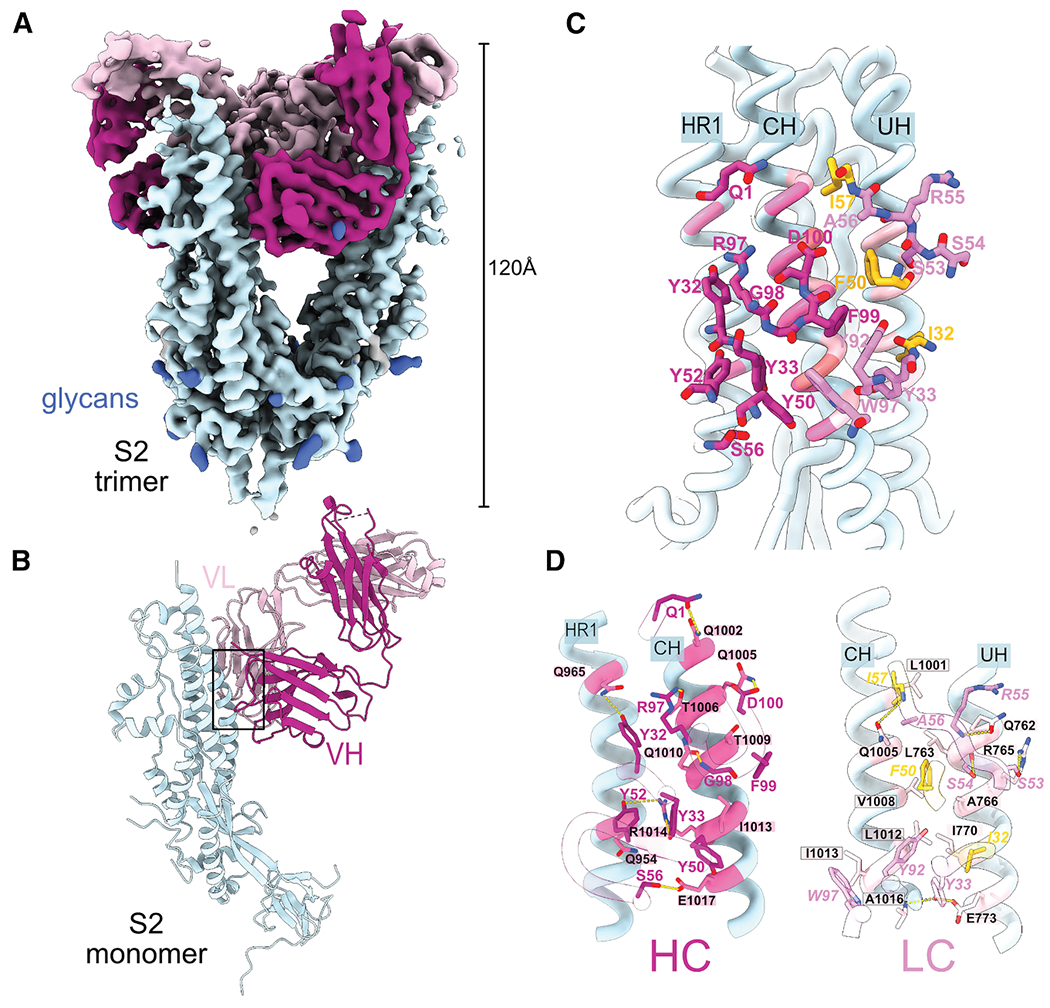
Cryo-EM structure of M15 in complex with S2 (A) Cryo-EM map of the trimeric S2-M15 Fab complex, highlighting S2 (light blue), M15 heavy chain (magenta), and M15 light chain (pink). Glycan densities are displayed in royal blue. (B) Cartoon representation of the S2 protomer-M15 Fab complex structure. (C) S2-M15 interface overview; region designated within black box in (B). S2 helices (ribbons) are labeled to designate the relative locations of heptad repeat 1 (HR1), central helix (CH), and upstream helix (UH). S2 residues are colored according to whether they form contacts with the correspondingly colored heavy chain (magenta) or light chain (pink). S2 residues contacting both heavy and light chains are colored coral. Convergent and clonotype-enriched mutations are colored yellow. (D) Close-up view of M15 heavy-chain (HC; left) and light-chain (LC; right) binding interfaces with S2. S2 residues are colored, as in (C), according to whether they form contacts with correspondingly colored heavy chain (magenta) or light chain (pink). Convergent and clonotype-enriched mutations are colored yellow.

Given that the convergent and clonotype-enriched SHMs enhanced the affinity of M15, we hypothesized that these mutations facilitate critical contacts between M15 and S2 from SARS-CoV-2. To address this, we obtained a 3.4 Å resolution cryogenic electron microscopy (cryo-EM) reconstruction of the M15 Fab bound to a stabilized prefusion SARS-CoV-2 spike S2 trimer^30^ (Figure S5; Table S4). The M15-S2 complex presented stoichiometric 3-fold symmetry, with one Fab unit bound to each protomer of the S2 trimer (Figure 4A). The structure disclosed a previously uncharacterized epitope situated on and around a segment of the S2 central helix (CH; Figure 4B). This epitope, which we refer to as the central interface (CI) epitope, is noteworthy for its location at a site of inter-protomer contacts in the canonical “closed” structure of the prefusion S2 homotrimer (Figure S6A). The CI epitope is accordingly occluded in the closed prefusion spike conformation, wherein central alpha helices assemble to form a triple-helix coiled coil. The epitope is thus only accessible in the open spike conformation,^31^ which differs markedly from the piriform S2 trimer observed in both the closed full-length spike complex and the unliganded stabilized prefusion S2 trimer.^30,32,33^ As the CI epitope is only accessible on the open S2, interaction between M15 and S2 favors the open S2 trimer, splaying protomers centrifugally outward from the 3-fold axis of the trimer (Figure S7). In agreement with our binding data demonstrating that M15 reactivity is limited to sarbecoviruses (Figure 1B), the contact residues on S2 are well conserved across sarbecoviruses but divergent across more distantly related HCoVs (Figures S6C and S6D).

We then analyzed the contacts of M15 Fab with the CI epitope to address our hypothesis that convergent and clonotype-enriched SHMs facilitate critical contacts with S2. The bound M15 Fab occupies 1315 Å^2^ of the buried surface area, with contacts spread across CH and nearby residues on two other alpha helices belonging to heptad repeat 1 (HR1) and the upstream helix (UH) (upstream of fusion peptide^32^; Figures 4C and 4D). Contacts were observed between both heavy and light Fab chains and CH (residues 1001–1016). Two of the convergent and clonotype-enriched SHMs, namely, S32I and T57I, along with the low convergence, clonotype-enriched SHM Y50F, indeed contributed to critical contacts with the CH and UH (Figures 2B, 2C, 4C, and 4D). These mutations replaced polar residues with hydrophobic ones, favoring thermodynamic stability. The mutated I57 residue in the light chain, which showed the highest convergence and clonotype enrichment (Figure 2C), also formed a ternary interaction with the CH residue Q1005, in combination with the heavy chain D100 via hydrogen bonds. Thus, the structural data validated that convergent and clonotype-enriched mutations help to drive the interaction between M15 and S2.

Additional contacts, inferred through proximity (<4Å) and side-chain functional group biochemical complementarity, were observed for heavy chain and light chain with HR1 and UH, respectively. Heavy-chain contacts (Q1, Y32, Y33, Y50, Y52, S56, R97, G98, F99, and D100) primarily comprise polar interactions with CH (1002–1017). Two contacts (Q1 and Y52) occur between the heavy chain and HR1 residues 961 and 965. Light-chain contacts are spread between CH (LC Y33, F50, A56, I57, Y92, G93, and W97) and UH (I32, Y33, F50, S53, S54, and R55). Light-chain residues support an abundance of hydrophobic interactions in addition to the convergent and clonotype-enriched SHMs (I57, F50, I32, Y92, and W97) and spatial complementarity with a correspondingly hydrophobic cleft formed between CH and UH (S2 residues Y756, F759, L763, A766, I770, L1001, L1004, V1008, L1012, I1013, A1015, and A1016; Figure S6B). An additional ternary interaction occurs at CH residue 1013 via hydrophobic contacts with heavy and light chains (Figure 4C). This clasping of CH appears to be enhanced by inter-Fab chain hydrogen bonds (LC Y37-HC F99; LC Q39-HC Q39; Figure S8). Having demonstrated that M15, aided by convergent and clonotype-enriched SHMs, binds to a previously uncharacterized epitope on S2, we next sought to investigate how antibodies targeting this epitopic region are shaped by SARS-CoV-2 infection or vaccination.

### Serum antibody levels targeting the CI epitope rise with repeated SARS-CoV-2 vaccinations

**Figure 5.**
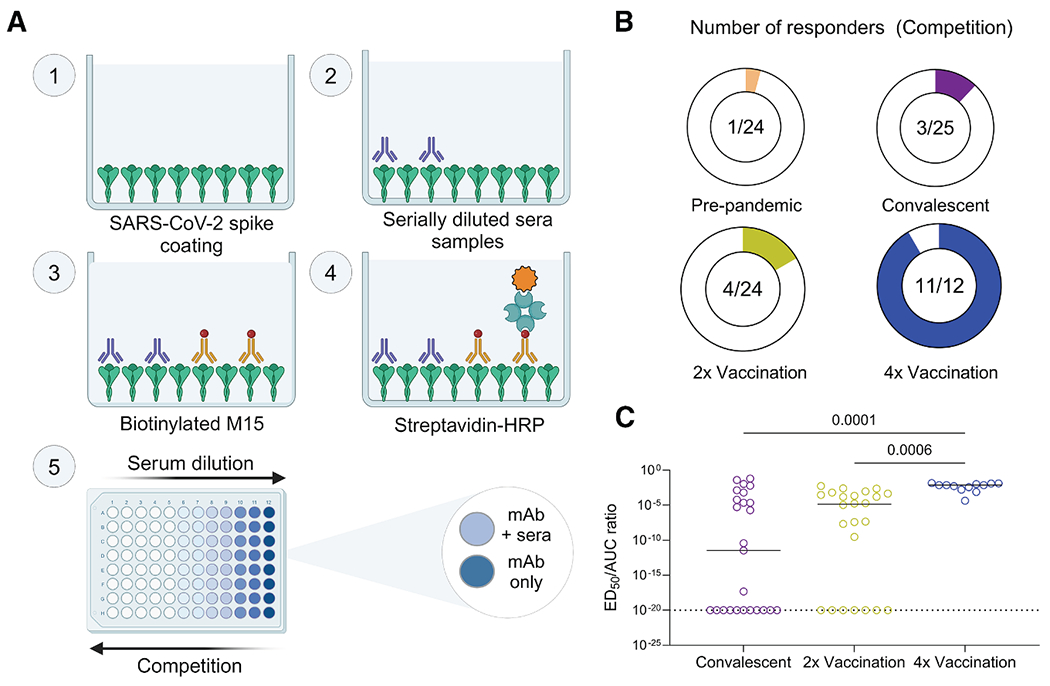
Prevalence of sera antibodies targeting the S2 central interface epitope in SARS-CoV-2-infected and vaccinated individuals (A) Schematic workflow of the protocol employed for the serum competition ELISA, created with BioRender. (B) Proportion of responders among the samples tested in a serum competition ELISA between M15 (biotinylated) and human sera from pre-pandemic samples (*n* = 24) or SARS-CoV-2 convalescent samples (*n* = 25), two doses of SARS-CoV-2 vaccination (*n* = 24), or four doses of SARS-CoV-2 vaccination (*n* = 12). Responders are identified as individuals showing competition ED_50_ values greater than 1. (C) The ratio of competition ED_50_ to anti-spike serum AUC values is depicted across individuals from the convalescent, vaccination (2×), and vaccination (4×) exposure groups. ED_50_/AUC values less than 10^−20^ were set to a detection limit of 10^−20^. Comparisons between exposure groups were performed using the Kruskal-Wallis test followed by Dunn’s multiple comparison correction. All serum competition ELISAs and serum ELISAs were performed in duplicates, and the ED_50_ values and AUCs were calculated as geometric means of the replicates. Horizontal bars represent the median of ED_50_/AUC ratios.

We assessed the extent of competition between the M15 mAb and sera of SARS-CoV-2 convalescent or vaccinated individuals to estimate and compare the prevalence of antibodies targeting the CI epitope (Figure 5A). Human sera samples (cohort 3) were obtained from four exposure groups: pre-pandemic (*n* = 24), SARS-CoV-2 convalescent (*n* = 25), two doses of SARS-CoV-2 vaccination (2× vaccination; *n* = 24), and four doses of SARS-CoV-2 vaccination (4× vaccination; *n* = 12; Tables S1 and S4). Individuals in the 4× vaccination group exhibited the highest competition with M15, with 91.7% (11 out of 12) classified as responders (median effective dilution (ED_50_) > 1; Figures 5B and S9A). In contrast, only one individual (4.16%) from the pre-pandemic group was classified as a responder (Figures 5B and S9A). CI epitope competition in the convalescent and 2× vaccination groups was low, with only 12% (3/25) and 16.6% (4/24) responders, respectively (Figures 5B and S9A).

We then investigated whether higher serum competition with M15 was a mere consequence of higher anti-spike serum antibody titers or if the proportion of serum antibodies targeting this epitopic region increases with multiple vaccinations (Figure S9B). To answer this, we normalized the extent of competition (i.e., ED_50_) with the serum antibody titers calculated as the area under the curve (AUC). While the extent of competition correlated positively with the serum antibody titers (ED_50_ vs. AUC Spearman correlation coefficient (r_s_) = 0.6042, *p* < 0.001; Figure S9C), the 4× vaccination group still showed significantly higher normalized competition compared with the 2× vaccination (Kruskal-Wallis test; *p* = 0.0006) and convalescent (*p* = 0.0002) groups (Figure 5C). This indicates that the proportion of serum antibodies targeting the CI epitope increases with each vaccine dose.

## DISCUSSION

SHMs acquired within a V gene exert their functional impact within the broader context of the clonotype, enhancing the affinity of the entire BCR specifically for its target antigen. A study by Tan and colleagues indeed demonstrated that an affinity-enhancing SHM, Y58F, in antibodies using the *IGHV3-53/66* genes was associated with antibodies presenting shorter CDR3 regions (fewer than 15 amino acids in length) instead of longer CDR3s, suggesting that the convergent acquisition of paratopic features, including SHMs, may contribute to increased affinity to the antigen. Thus, the fact that the same V gene can be used by antibodies with different specificities raises an important question: are affinity-enhancing SHMs specific to the antibody clonotype in which they increase affinity? Or do they appear at similar frequencies in antibodies with different epitope specificities using the same V gene?

We addressed these questions by discovering and characterizing a human public antibody, M15, elicited upon SARS-CoV-2 exposure, which uses the *IGHV4-59*/*IGHJ4* and *IGKV3-20*/*IGKJ1* heavy- and light-chain V/J genes, respectively. Notably, previous studies have identified public clonotypes using the same V and J genes and CDR3 sequences as M15 that are also elicited upon SARS-CoV-2 exposure.^3,25,34^ Using 147 M15-like sequences, we identified five convergent and clonotype-enriched SHMs exclusively in the light chain. Reversion of three of these SHMs to their germline residues resulted in a substantial decrease in binding affinity, suggesting that these convergent and clonotype-enriched SHMs drive affinity maturation of M15. The minimal change in affinity following individual S32I, T57I, and G51A reversions suggests that binding interactions are driven by the synergistic effect of these linked mutations. The role of G51A in the triple revertant loss of affinity remains particularly puzzling, as our cryo-EM structure suggests that A51 is not a direct contact residue. Taken together with the observed linkage between G51A and T57I, our findings highlight the importance of analyzing individual antigen-contacting residues within the broader context of interactions between SHMs, which may drive their concomitant acquisition during affinity maturation.^35–37^

Affinity-enhancing SHMs have previously been shown to be convergently selected in public antibodies,^3,15^ raising the question of how much the concept of clonotype enrichment helps narrow down affinity-enhancing SHMs, beyond simple convergence. This is particularly evident when comparing the frequencies of heavy-chain SHMs G27D and S31N (Figure 2C) to light-chain SHMs Y50F and S32I. While all four SHMs exhibit similar levels of convergence, Y50F and S32I show significant clonotype enrichment. In contrast, G27D and S31N have convergence rates comparable to background frequencies. Structural analysis further supported this distinction, revealing that only Y50F and S32I are involved in contacts, while G27D and S31N are not (Figures 4C and 4D), reinforcing that clonotype enrichment is a characteristic feature of affinity-enhancing SHMs. Because computing clonotype enrichment requires estimates of the background frequency of convergent SHMs, our framework can be integrated with tools such as ARMADiLLO,^38^ which estimates the likelihood of SHM occurrence at specific residues within antibody variable regions, and could facilitate the identification of affinity-enhancing SHMs in the context of other antigens.

Our structural analysis also revealed that M15 recognizes a previously unappreciated CI epitope accessible on the open configuration of S2. We observed a vaccine dose-dependent increase in sera antibodies targeting the S2 CI epitope, with individuals who received four SARS-CoV-2 vaccine doses showing a higher proportion of these antibodies than individuals who received two doses or those convalescent from SARS-CoV-2 infection. Further investigation is needed to determine if this is indicative of a shift in immunodominance upon multiple exposures to SARS-CoV-2 and if other antibodies (if any) targeting this epitope are also non-protective like M15.

In conclusion, our study emphasizes that affinity maturation of public antibodies is driven by convergent and clonotype-enriched SHMs and reveals a previously undescribed CI epitope in S2 of sarbecoviruses. Identifying other public antibodies, not only against viruses but also against other pathogens, could provide valuable mechanistic insights into affinity maturation. This could further inform the design of immunogens that drive favorable affinity maturation pathways and elicit affinity-enhancing SHMs that improve the neutralization potency against a broader range of pathogens.

### Limitations of the study

While our study demonstrates that convergent and clonotype-enriched SHMs play a crucial role in affinity maturation, it is currently limited to the M15 clonotype. Further investigation is needed to determine whether this phenomenon extends to other clonotypes. Although the SHM G51A was both convergent and clonotype enriched, it did not directly mediate interactions with the CI epitope. Its contribution to the affinity of M15 could not be independently verified, as the corresponding single mutant did not exhibit a significant reduction in binding affinity compared with M15. Nonetheless, the observed co-occurrence of G51A with T57I suggests a potential interaction between these two convergent and clonotype-enriched mutations. The contribution of Y37F to M15’s affinity could not be assessed, as this mutation was absent in the original M15 sequence. Notably, linkage analysis revealed a negative association between Y37F and S32I in donor 1 (Figure S4A), and between Y37F and both S32I and T57I in donor 2 (Figure S4B), indicating a potential antagonistic relationship between Y37F and these other affinity-enhancing SHMs. However, due to the limited number of M15-like sequences available, we are unable to draw definitive conclusions. Finally, since we identified M15-like antibodies following vaccination with mRNA vaccines and ChAdOx1 nCoV-19 vaccine (an adenoviral vector-based vaccine), we cannot determine whether their emergence is specific to a vaccine platform, an effect that warrants further investigation.

## RESOURCE AVAILABILITY

### Lead contact

Further information and requests for resources and reagents should be directed to and will be fulfilled by the lead contact, Camila H. Coelho (camila.coelho@mssm.edu).

### Materials availability

All reagents will be made available upon request after completion of a materials transfer agreement.

### Data and code availability

Heavy- and light-chain nucleotide sequences from plasmablasts in cohort 1 (BioProject: PRJNA1134144) and plasmablasts and memory B cells in cohort 2 (GenBank: PV911686–PV915173) have been deposited on the National Center for Biotechnology Information (NCBI) portal. The EM maps have been deposited in the Electron Microscopy Data Bank (EMDB) under accession code EMD-48507 and the accompanying atomic coordinates in the Protein Data Bank (PDB) under accession code 9MPW. Code used for the mutation analysis has been deposited on GitHub (https://doi.org/10.5281/zenodo.15691797). Additional data supporting the findings can be found in the supplemental information.

## STAR★METHODS

### EXPERIMENTAL MODEL AND STUDY PARTICIPANT DETAILS

#### Human samples

##### Cohort 1

Samples were collected from three individuals who received either Pfizer’s Comirnaty or Moderna’s Spikevax XBB.1.5 mRNA-based vaccine and two individuals who received Novavax’s protein-based XBB.1.5 vaccine as described in our previous study.^22^ Plasma-blasts were sorted 6–7 days post-vaccination as detailed in Fantin et al., 2024.

##### Cohort 2

Blood samples were collected from COVID-19 infection naive individuals immunized with the COVID-19 mRNA vaccine (*n* = 19). A longitudinal study was conducted where blood was collected one month after the second dose, six months after second dose, and 10 days after the third dose. The vaccination type, vaccination dates and history of COVID-19 were collected from each participant. All procedures involving human samples were approved by the Massachusetts General Brigham (MGH) Institutional Review Board (Protocol #:2020P000837). Informed consent was collected from participants.

##### Cohort 3

The human subjects’ samples used in this paper are sourced from two IRB approved observational research study protocols (STUDY-16-01215/IRB-16-00971 and STUDY-20-00442/IRB-20-03374) that collect samples before and after viral antigen exposure. All study participants provided informed consent for participation in research prior to data or sample collection. All human subjects research is reviewed and approved by the Program for the Protection of Human Subjects at the Icahn School of Medicine at Mount Sinai. For set up and optimizations of the experimental work, additional samples were used from enrolled participants who provided samples prior to the SARS-CoV-2 pandemic.

Clinical metadata annotating the biospecimen at the time of collection was compiled through self-reported data from participants. Sera collected under the IRB approved protocols listed above were leveraged in this analysis. 24 sera samples from participants prior to SARS-CoV-2 vaccine approval (SARS-CoV-2 antibody negative), 25 sera samples from participants prior to SARS-CoV-2 vaccination but with evidence of a SARS-CoV-2 infection, 24 sera samples from participants who had completed primary SARS-CoV-2 immunization (2 doses of the mRNA SARS-CoV-2 vaccine) and 12 sera samples from participants who received two additional vaccinations after primary immunization were collected.

Demographics, COVID-19 vaccination, and SARS-CoV-2 infection information for Cohorts 1, 2, and 3 are summarized in Table S1.

#### Cells and viruses

Vero E6 cells expressing transmembrane protease serine 2 (TMPRSS2; BPS Biosciences, 78081) were maintained in Dulbecco’s modified Eagle medium (DMEM; Gibco) supplemented with 10% fetal bovine serum (FBS), 1% minimum essential medium (MEM) amino acids solution (Gibco, 11130051), 100 units/mL penicillin and 100 μg/mL streptomycin (Gibco, 15140122), 3 μg/mL puromycin (InvivoGen, ant-pr-1), and 100 μg/mL normocin (InvivoGen, ant-nr-05). Expi293 cells were maintained in Expi293 expression media (Gibco, A1435101).

SARS-CoV-2 WA1/2020 viral stocks obtained from BEI Resources, and SARS-CoV-2 XBB.1.5 (hCoV-19/USA/NY-MSHSPSP-PV76648/2022) and SARS-CoV-2 JN.1 (hCoV-19/USA/NYMSHSPSP-PV96109/2023) viral stocks obtained from the Personalized Virology Initiative (PVI) at Mount Sinai, were confirmed through sequencing and quantified using the 50% tissue culture infectious dose (TCID_50_) method.

### METHOD DETAILS

#### Plasma and peripheral blood mononuclear cell (PBMC) isolation

Blood samples from Cohort 2 participants was collected in EDTA tubes. Blood was centrifuged at 300 g for 10 min. Plasma was collected from the top layer and further centrifuged at 1000 g for 10 min for the removal of debris. Plasma was divided into aliquots and stored in −80°C. PBMCs were collected from the lower layer of the initial centrifuge step. Ficoll-Paque (Cytiva) was added to the lower layer for PBMC separation at 2200 rpm for 20 min. PBMCs were washed with PBS and resuspended in FBS containing 10% dimethyl sulfoxide (DMSO). Samples were aliquoted and stored at −80°C and liquid nitrogen.

#### Flow cytometry and single-cell sorting

PBMCs stored at −80°C were thawed at 37°C and transferred to warm RPMI 1640 media (Gibco) supplemented with 10% FBS (Cytiva). B cells were enriched via positive selection of with anti-CD19 beads (Miltenyi). As previously described,^51^ spike-positive memory B cells were sorted (BD FACSAria Fusion) into 96-well plates. In brief, enriched B cells were stained with SARS-CoV-2 spike containing a FLAG tag (Genscript). Following primary incubation, the enriched B cells were stained with allophycocyanin (APC)-conjugated anti-Flag and phycoerythrin (PE)-conjugated anti-Flag for double-positive sorting. DAPI^−^ CD20^+^ CD19^+^ IgM^−^ IgD^−^ IgG^+^ CD27^+^ Spike^+^ cells for memory B cell, and DAPI^−^ CD20^−^ CD19^+^ CD38^hi^ CD27^+^ cells for plasmablasts were single cell sorted into 96-well PCR plates containing lysis buffer (4 μL of 0.5× PBS with 10 mM dithiothreitol and 4U of RNAseOUT). Plates were stored at −80°C.

#### Bioinformatic analysis of B cell receptor (BCR) sequencing data and database mapping

Heavy and light chain single-cell V(D)J sequences were obtained from plasmablasts from Cohort 1 after running 10× Genomics Cell Ranger v7.1.0 on FASTQ files generated via single-cell RNA (sc-RNA) sequencing, as described by Fantin and colleagues.^22^ For Cohort 2, as previously described,^51^ RNA from single cell sorted spike positive memory B cells were reverse transcribed into cDNA for amplification of IgH, Igκ, and Igƛ genes. In brief, cDNA was amplified using two semi-nested PCRs.^52^ Sanger sequencing was performed on the PCR products from the second amplification. Cells with unique and productive heavy and light chains were used for further analysis. Each paired sequence was annotated with its V(D)J genes using the AssignGenes and ParseDb functions in the changeo module of the Immcantation framework.^42^ Paired VH/VL sequences from the CoV-AbDab and OAS databases were downloaded from the version updates on November 14, 2023, and June 21, 2024, respectively.^26,43^ For CoV-AbDab, sequences with undefined V or J genes or CDR3 sequences for heavy and light chains and those from species other than humans were filtered out. Sequence-based mapping to databases was performed in R, as follows: a sequence from the cohort was mapped to a sequence in the database if i) they had the same V and J gene annotations in both heavy and light chains and ii) they had a CDR3 amino acid similarity greater than or equal to 70% in both heavy and light chains.^25^ The same matching criteria were followed while mapping M15 from Cohort 1 to Cohort 2.

#### Sequence analysis

M15-like VH and VL amino acid sequences were aligned using the COBALT multiple alignment tool (https://www.ncbi.nlm.nih.gov/tools/cobalt/re_cobalt.cgi). Mutation frequencies within clonal lineages and other M15-like sequences were assessed from FASTA-formatted files using R and Perl.

#### mAB production and purification

The M15 mAb was produced based on the protocol described by Fantin and colleagues.^22^ Briefly, the VH and VK genes of M15 were cloned into pTwist mammalian expression vectors. Expi293 cells (25- to 200-mL cultures) were transfected with the heavy- and light-chain-expressing vectors at a 1:2 ratio by concentration, along with Expifectamine (transfection reagent; Thermo Fisher Scientific), following the protocol described in the lipid-based Thermo Fisher Expi293 Expression System Kit. The cells were incubated in a shaker flask at 37°C for 18–22 h at 125 rpm. Enhancers 1 and 2 were added to the culture the next day, followed by further incubation for 5–6 days. Cells were then pelleted down by centrifugation and filtered (0.45 μm). The supernatant was mixed with protein A agarose resin (Gold Biotechnology) in a rotary shaker for 12 h at 4°C and then loaded onto Poly-Prep chromatography columns (Bio-Rad Laboratories, Inc.) and eluted with glycine in phosphate-buffered saline (PBS), followed by neutralization with 1 M Tris pH 8 and 5 M NaCl. Purified antibodies were concentrated and stored at 4°C.

#### ELISA

For ELISA, 96-well Immulon 4 HBX plates (Thermo Scientific) were coated with 50 μL of the recombinant protein at 2 μg/mL and were stored at 4°C overnight. Wells were then washed three times with phosphate-buffered saline (3) containing 0.1% Tween 20 (PBST) and blocked with 100 μL PBST supplemented with 3% milk for 1 h at room temperature. Antibodies were diluted 3-fold from a starting concentration of 30 μg/mL, and sera were diluted 2-fold from a starting concentration of 1:25, all in PBST supplemented with 1% milk in a separate 96-well round-bottomed plate. For the vaccination (4×) sera samples, a starting dilution of 1:50 was used. After the blocking solution was discarded, 100 μL of the diluted antibodies or sera was added to the corresponding wells, followed by incubation for 1 h (antibodies) or 2 h (sera) at room temperature. The antibody or sera solutions were then discarded, and the wells were washed three times with PBST. Next, 100 μL of mouse anti-human IgG antibody conjugated to horseradish peroxidase (Sigma, A0293) at a 1:3000 dilution was added to each well, followed by incubation for 1 h at room temperature. Plates were washed three times with PBST and developed by adding 100 μL of o-phenylenediamine dihydrochloride (OPD; Sigma-Aldrich) in phosphate citrate buffer (PCB; Sigma-Aldrich) with 0.04% hydrogen peroxide for 10 min. The reaction was stopped by adding 50 μL of 3 M hydrochloric acid (HCl; Fisher). The optical density (OD) was read for each well using a Synergy 4 (BioTek) plate reader at a wavelength of 490 nm. AUC values were computed in GraphPad Prism (v10.2.3), setting the average plus five times the standard deviation of the OD of blank wells as the baseline.

#### Biolayer interferometry (BLI)

Anti-human IgG Fc biosensors (Sartorius; 18–5001) were hydrated in 10 nM Tris, 150 mM NaCl, and 0.1% Tween buffer for 10 min. Then, 2 μg/mL M15 diluted in the same buffer was immobilized on a biosensor, and purified recombinant S2 protein was flowed at different concentrations, starting from 166.7 nM to 16,666.7 nM, using the ForteBio Sartorius BLItz System. The steps involved are briefly described as follows: i) Baseline: the biosensor was initialized at baseline by dipping it in buffer for 30 s, ii) Loading: 4 μL of the antibody (M15) at 2 μg/mL was loaded onto the biosensor for 90 s, iii) Baseline: the biosensor was dipped back in buffer for 30 s, iv) Association: 4 μL of the antigen (S2) at different concentrations was loaded onto the biosensor for 60 s, and v) Dissociation: the biosensor was dipped back into the buffer to let the antigen dissociate for 90 s. A control run with buffer loaded instead of the antibody was performed to confirm specific binding. The R_max-apparent_ values for association, with the baseline signal before association subtracted, were plotted against the S2 concentrations, and the K_D_ was determined using a non-linear fit for one-site specific binding in GraphPad Prism (v10.2.3).

#### Serum competition ELISA

For serum competition ELISA, 96-well Immulon 4 HBX plates (Thermo Scientific) were coated with 50 μL of the recombinant spike protein at 2 μg/mL and stored at 4°C overnight. Wells were then washed three times with PBST and blocked with PBST supplemented with 3% milk for 1 h at room temperature. Serum was diluted 2-fold from a starting dilution of 1:25 in PBST supplemented with 1% milk in a separate 96-well round-bottomed plate. After the blocking solution was discarded, 100 μL of the diluted serum was added to the corresponding wells, followed by incubation for 2 h at room temperature. For the mAb-only positive control, PBST supplemented with 1% milk was added instead of serum. After incubation, the serum solutions or control solution was discarded, and the wells were washed three times with PBST. For each serum sample, 100 μL of biotinylated M15 antibody, or an mpox-specific antibody as a negative control, was added to each dilution in duplicates at two times the minimum binding concentration of M15 with spike protein (quantified by ELISA), followed by incubation for 2 h at room temperature. The antibody solutions were then discarded, and the wells were washed three times with PBST. Next, 100 μL of streptavidin conjugated to horseradish peroxidase (Pierce, 21130) at a 1:1000 dilution was added to each well, followed by incubation for 1 h at room temperature. Plates were washed three times with PBST and developed by adding 100 μL of OPD (Sigma-Aldrich) in PCB (Sigma-Aldrich) with 0.04% hydrogen peroxide for 10 min. The reaction was stopped by adding 50 μL of 3 M HCl (Fisher). The absorbance was read for each well using a Synergy 4 (BioTek) plate reader at a wavelength of 490 nm. Competition ED_50_ values were computed by fitting the OD-dilution curves with the ‘Absolute IC_50_’ function in GraphPad Prism (v10.2.3).

#### Neutralization assay

Microneutralization assays were performed as previously detailed.^39^ In brief, Vero E6 TMPRSS2 cells were seeded in 96-well tissue culture plates (Corning, 3340) at a density of 1 × 10^4^ cells per well. After 24 h, mAbs were diluted to an initial concentration of 30 μg/mL in 1× MEM with 2% FBS and then serially diluted 1:3 to achieve a final concentration of 0.041 μg/mL. An 80-μL portion of the mAb dilution was mixed with 80 μL of SARS-CoV-2 diluted to 10^4^ TCID_50_/mL, and this mixture was incubated at room temperature for 1 h. Following incubation, 120 μL of the virus/mAb mixture was used to infect the cells for 1 h. The inoculum was subsequently removed and replaced with 100 μL of 1× MEM containing 2% FBS and 100 μL of antibody dilution. The cells were then incubated at 37°C in a 5% CO_2_ environment for 48 h before fixation with 10% paraformaldehyde (Polysciences) for 24 h. After fixation, the paraformaldehyde was discarded, and cells were permeabilized by adding 100 μL of PBS containing 0.1% Triton X-100 (Fisher) for 15 min at room temperature. Once permeabilization was complete, the Triton X-100 solution was removed, and cells were blocked with 100 μL of 3% non-fat milk (Life Technologies) diluted in PBS. Finally, cells were stained for SARS-CoV-2 nucleoprotein using mAb 17C7, as previously described.^39^

#### Mice protection studies

Animal studies were performed in accordance with the guidelines set forth by the Icahn School of Medicine at Mount Sinai’s Institutional Animal Care and Use Committee (IACUC) and under approved protocols reviewed by the IACUC. The 50% lethal dose (LD_50_) was determined for WA1/2020, XBB.1.5, and JN.1 by infecting 6- to 8-week-old hACE2-K18 mice (Jackson Laboratory) with serial dilutions of the virus, ranging from 10^5^ to 5 plaque-forming units (PFU) in sterile PBS. For the protection studies, mice received an intraperitoneal injection of mAb at a dosage of 10 mg/kg, diluted in 100 μL of sterile PBS. Two hours post-treatment, mice were anesthetized using 0.15 mg/kg ketamine and 0.03 mg/kg xylazine, both diluted in water for injection (WFI, Gibco), and intranasally infected with a 3xLD_50_ dose of SARS-CoV-2 WA1/2020, XBB.1.5, or JN.1 diluted in 50 μL of sterile PBS. Mice were monitored for weight loss for 14 days following infection, and any animals that lost more than 25% of their body weight were humanely euthanized. The influenza A virus antibody CR9114 served as a negative control.^40^

#### Cryogenic electron microscopy (Cryo-EM) sample preparation

SARS-CoV-2 stabilized S2 domain was incubated with M15 Fab at 2.5 mg/mL at a molar ratio of 1.5:1 Fab:S2 for 20 min at 4°C. Immediately before grid preparation, fluorinated octyl maltoside was added to the pre-formed complex at 0.02% w/v final concentration. Then, 3-μL aliquots were applied to UltrAuFoil gold R1.2/1.3 grids (Quantifoil) and subsequently blotted for 6 s at blot force 1 and then plunge-frozen in liquid ethane using an FEI Vitrobot Mark IV. Grids were imaged on a Titan Krios microscope operated at 300 kV and equipped with a Gatan K3 Summit direct detector. 8,134 movies were collected in counting mode at 16e–/pix/s at a magnification of 105,000, corresponding to a calibrated pixel size of 0.826 Å. Defocus values were from −0.5 to −1.5 μm.

#### Cryo-EM data processing

Movies were aligned and dose-weighted using MotionCorr2.^45^ Contrast transfer function estimation was done in cryoSPARC v4.6 using Patch CTF, and particles were picked with cryoSPARC’s blob picker. The selected particles were extracted with a box size of 512 pixels, with ~4Å binning, and subjected to a 2D classification. Particles from well-defined 2D classes were then refined through a second round of 2D classification. The resulting 2D class averages were used as templates for template-based particle picking, followed by multiple rounds of 2D classification, yielding 91,579 particles. An ab initio model was generated from 91,597 particles at ~6 Å/pixel, using five classes. The best-resolved class was selected for 3D classification in cryoSPARC. Particles from the most promising 3D class were re-extracted at full box size and subjected to non-uniform refinement. At this stage, local CTF refinement and reference-based motion correction were applied. The protomer exhibiting the best-defined Fab density was further refined using local refinement with a soft mask encompassing the S2 region and Fab, resulting in a final local map at 3.37 Å resolution. The final map was sharpened using CryoSPARC’s map sharpening tool. The protomer with the best Fab volume was subjected to local refinement with a soft mask encompassing S2 and Fab, yielding a final local map at 3.37Å. The CryoSPARC sharpening tool was implemented to obtain a sharpened map.

#### Atomic model building and refinement

The Cryosparc-sharpened map was used for model building with ModelAngelo^46^ and then manually built using COOT.^47^ N-linked glycans were built manually in COOT using the glyco extension. The model was then refined in Phenix^48^ using real-space refinement and validated with MolProbity.^49^ The structural biology software was compiled and made available through SBGrid.^53^

#### Clonal lineage inference

Clonal lineages using paired heavy and light chain M15-like sequences from OAS and CoV-AbDab were constructed, and the UCA and intermediate sequences were inferred using Cloanalyst.^50^

### QUANTIFICATION AND STATISTICAL ANALYSIS

Statistical analysis was performed on GraphPad Prism and *p*-values were generated using a chi-squared test with Yates’ correction in Figure 2C, the Kruskal-Wallis test followed by Dunn’s multiple comparison correction in Figures 5C, S9A, and S9B and Spearman correlation test in Figure S9C.

#### Linkage analysis

For each clonal lineage, association or linkage between each pair of convergent mutations was assessed by constructing a contingency table of co-occurrence of the two mutations. The odds ratio was computed by running the fisher.test() function on the contingency table using the R package ‘stats’.

## Supplementary Material

1

Supplemental information can be found online at https://doi.org/10.1016/j.celrep.2025.116122.

## Figures and Tables

**Table 1.** Sources of M15-like antibody sequences

Condition (cohort)	Total number of sequences	M15-like sequences
XBB.1.5 mRNA/protein-based vaccination (cohort 1)	603	1
CoV-AbDab	11,120	17
SARS-CoV-2 mRNA vaccination (ancestral strain; cohort 2)	1,794	7
SARS-CoV-2 infection (OAS)	774,147	122
Healthy (OAS)	568,139	0
Obstructive sleep apnea (OAS)	3,327	0
Tonsillitis (OAS)	17,611	0
Tonsillitis/obstructive sleep apnea (OAS)	10,133	0
HIV-1 (OAS)	5,547	0
Multiple sclerosis (OAS)	179,468	0
Influenza virus infection/vaccination	3,734	0
Malaria vaccination	193	0

The database or cohort source for each condition is mentioned in parentheses. Sequences from influenza virus infection/vaccination and malaria vaccination were compiled in-house from 26 independent studies (described in Table S3).

**Table:** KEY RESOURCES TABLE

REAGENT or RESOURCE	SOURCE	IDENTIFIER
Antibodies		
Goat anti-human IgG-alkaline phosphatase (AP)	Southern Biotech	Cat# 2040-04
Human CD19 MicroBeads	Miltenyi Biotec	Cat# 130-050-301; RRID: AB_2848166
Anti-flag-APC	BioLegend	Cat# 637307; RRID:AB_2561496
Anti-flag-PE	BioLegend	Cat# 637310; RRID:AB_2563148
Anti-His-PE	BioLegend	Cat# 362603; RRID:AB_2563634
Anti-human-IgG-PerCP-Cy5.5	BioLegend	Cat# 410710; RRID:AB_2565788
Anti-human-CD27-APC-Cy7	BioLegend	Cat# 356424; RRID:AB_2566773
Anti-human-CD19-BV510	BioLegend	Cat# 302242; RRID:AB_2561668
Anti-human-IgD-FITC	BioLegend	Cat# 348206; RRID:AB_10612567
Anti-human-IgM-BV605	BioLegend	Cat# 314524; RRID:AB_2562374
Anti-human-IgG-Alexa Fluor 647	Thermo Fisher Scientific	Cat# A-21445; RRID:AB_2535862
HRP-conjugated mouse anti-human IgG secondary antibody	Sigma-Aldrich	Cat# RABHRP3
mAb 1C7C7	Mount Sinai, Carreno et al.^39^	ZMS1075
mAb CR9114	Dreyfus et al.^40^	N/A
Bacterial and virus strains		
SARS-CoV-2 WA1/2020 virus	BEI Resources	NR-52281
SARS-CoV-2 XBB.1.5 (hCoV-19/USA/NY-MSHSPSP-PV76648/2022)	PVI, Mount Sinai	PV76648
SARS-CoV-2 JN.1 (hCoV-19/USA/NYMSHSPSP-PV96109/2023)	PVI, Mount Sinai	PV96109
Biological samples		
Plasmablasts from XBB.1.5 mRNA and protein-based vaccinated donors	Fantin et al.^22^	N/A
Plasmablasts and memory B cells from SARS-CoV-2 mRNA vaccinated donors	Wesemann laboratory	N/A
Serum from SARS-CoV-2 vaccinated and convalescent donors (Cohort-3)	Simon laboratory	N/A
Chemicals, peptides, and recombinant proteins		
SARS-CoV-2 WA1/2020 spike protein	Krammer laboratory	N/A
229E HCoV spike protein	Krammer laboratory	N/A
HKU1 HCoV spike protein	Krammer laboratory	N/A
OC43 HCoV spike protein	Krammer laboratory	N/A
NL63 HCoV spike protein	Krammer laboratory	N/A
MERS-CoV spike protein	Krammer laboratory	N/A
SARS-CoV-2 spike protein	Krammer laboratory	N/A
SARS-CoV-2 WA1/2020 S2 protein	Bajic laboratory	N/A
SARS-CoV-2 WA1/2020 RBD protein	Krammer laboratory	N/A
SARS-CoV-2 WA1/2020 NTD protein	Bajic laboratory	N/A
SARS-CoV-2 spike protein (sorting)	Genscript	Cat# Z03481
10× phosphate buffered saline (PBS)	Boston BioProducts	Cat# BM-220-10XS
Glycine	Sigma-Aldrich	Cat# G7126
Zinc chloride	Sigma-Aldrich	Cat# 793523
Magnesium chloride	Sigma-Aldrich	Cat# M0250
Tween 20	Sigma-Aldrich	Cat# P1379
Ficoll-Paque PLUS	Cytiva	Cat# 17144003
4′,6-diamidino-2-phenylindole, dihydrochloride (DAPI)	Thermo Fisher Scientific	Cat# D1306
Dithiothreitol (DTT)	Thermo Fisher Scientific	Cat# R0861
RNaseOUT	Thermo Fisher Scientific	Cat# 10777-019
Random hexamer primer	Thermo Fisher Scientific	Cat# FERSO142
10 mM dNTPs	Promega	Cat# U1515
IGEPAL CA-630	Sigma-Aldrich	Cat# I8896
SuperScript III reverse transcriptase	Thermo Fisher Scientific	Cat# 18080085
HotStarTaq DNA polymerase	QIAGEN	Cat# 203205
Protein A agarose resin	Gold Biotechnology	Cat# P-400-5
*o*-phenylenediamine dihydrochloride (OPD)	Sigma-Aldrich	Cat# P4664
Phosphate Citrate Buffer (PCB)	Sigma-Aldrich	Cat# P4809
Hydrochloric Acid	Fisher	Cat# S25856
HRP-conjugated streptavidin	Pierce	Cat# 21130
Expi293^™^ expression media	Gibco	Cat# A1435101
Dulbecco’s modified eagle medium (DMEM)^™^	Gibco	Cat# 11995065
Minimum essential medium (MEM) amino acids solution	Gibco	Cat# 11130051
Penicillin-streptomycin	Gibco	Cat# 15140122
Puromycin	Invivogen	Cat# ant-pr-1
Normocin	Invivogen	Cat# ant-nr-5
10% Formaldehyde	Polysciences	Cat# 04018-1
Water for injection (WFI)	Gibco	Cat# A12873-01
Fluorinated octyl maltoside	Anatrace	Cat# O310F
Critical commercial assays		
ExpiFectamine293^™^ transfection kits	Gibco	Cat# A14525
Pierce^™^ Antibody Biotinylation Kit	Pierce	Cat# 90407
Deposited data		
Paired heavy/light chain sequences (Cohort 1)	Fantin et al.^22^	BioProject: PRJNA1134144
Paired heavy/light chain sequences (Cohort 2)	This paper	GenBank: PV911686 - PV915173
Electron microscopy maps of M15 in complex with S2	This paper	EMDB: EMD-48507
CryoEM structure of M15 Fab in complex with S2	This paper	PDB ID: 9MPW
Code used for mutation analysis	This paper	GitHub: https://doi.org/10.5281/zenodo.15691797
Experimental models: Cell lines		
Expi293F^™^	Thermo Fisher	Cat# A14528; RRID:CVCL_D615
Vero.E6.TMPRSS2^™^	BPS Biosciences	Cat # 78081
Experimental models: Organisms/strains		
B6.Cg-Tg(K18-ACE2)2Primn/J mice	The Jackson Laboratory	Cat# 034860; RRID: IMSR_JAX:034860
Oligonucleotides		
Primer set for Ig PCR (Tiller et al., 2008)	IDT DNA	N/A
Recombinant DNA		
Plasmid pTwist mammalian expression vectors	Twist Biosciences	https://www.twistbioscience.com/
Software and algorithms		
IgBLAST	Ye et al.^41^	https://www.ncbi.nlm.nih.gov/igblast/
Cell Ranger (v7.1.0)	10× Genomics	https://www.10xgenomics.com/
Immcantation	Gupta et al.^42^	https://immcantation.readthedocs.io/en/stable/index.html#
CoV-AbDAb	Raybould et al.^43^	https://opig.stats.ox.ac.uk/webapps/covabdab/
Observed Antibody Space (OAS)	Olsen et al.^26^	https://opig.stats.ox.ac.uk/webapps/oas/
COBALT Multiple Sequence Alignment tool	Papadopoulos et al.^44^	https://www.ncbi.nlm.nih.gov/tools/cobalt/re_cobalt.cgi
RStudio (v4.2.1)	RStudio	https://posit.co/download/rstudio-desktop/
Perl	Programming Perl	https://www.perl.org/
GraphPad Prism (v10.2.3)	GraphPad	https://www.graphpad.com/
MotionCorr2	Zheng et al.^45^	N/A
CryoSPARC (v3.3.1)	Structura Biotechnology	https://cryosparc.com/
ModelAngelo	Jamali et al.^46^	N/A
COOT	Emsley et al.^47^	N/A
Phenix	Leibschner et al.^48^	N/A
MolProbity	Williams et al.^49^	N/A
SBGrid	SBGrid consortium	https://sbgrid.org/
Cloanalyst	Kepler et al.^50^	N/A
Others		
Anti-human IgG Fc biosensors	Sartorius	Cat# 18-5060
UltrAuFoil gold R1.2/1.3 grids	Quantifoil	Cat# TEM-Q350AR13A

## Highlights

- Clonotype-enriched SHMs are identified in the light chain of a public antibody, M15
- These light-chain SHMs enhance affinity of M15
- M15 targets a previously undescribed central interface S2 epitope in sarbecoviruses
- Serum antibodies targeting this epitopic region are increased after multiple vaccinations
