# Supplementary material for: Clonotype-enriched somatic hypermutations drive affinity maturation of a public human antibody targeting an occluded sarbecovirus epitope
Source: Cell Rep. Author manuscript; Available in PMC 2025 Sep 29. (PMC12477886; doi:10.1016/j.celrep.2025.116122)
Supplement: 1 [file NIHMS2107281-supplement-1.pdf]

Cell Reports, Volume *44*

Supplemental information

Clonotype-enriched somatic hypermutations drive affinity maturation of a public human antibody targeting an occluded sarbecovirus epitope

Vishal N. Rao, Iden A. Sapse, Hallie Cohn, Duck-Kyun Yoo, Pei Tong, Jordan J. Clark, Bailey Bozarth, Yuexing Chen, Komal Srivastava, Gagandeep Singh, Florian Krammer, Viviana Simon, Duane R. Wesemann, Goran Bajic, and Camila H. Coelho

Supplementary Material

TABLES

Demographic	Cohort 1<br>n = 5	Cohort 2<br>n = 19	Cohort 3<br>n = 85
**Age groups (number of individuals)**			
18-29	0	3	21
30-39	1	9	23
40-49	2	4	18
50-59	1	1	14
60-69	1	1	4
70-79	0	1	5
**Sex at birth**	3M, 2F	6M, 13F	33M, 52F
**Previous immune history to SARS-CoV-2**	XBB.1.5 mRNA or protein-based vaccination, five to six previous doses of SARS-CoV-2 vaccination, none to two previous SARS-CoV-2 infection	Two or three doses of SARS-CoV-2 mRNA vaccination, no previous SARS-CoV-2 infection	Pre-pandemic (n = 24)<br>SARS-CoV-2 convalescent (n = 25)<br>Two doses of SARS-CoV-2 mRNA vaccination (n = 24)<br>Four doses of SARS-CoV-2 mRNA vaccination (n = 12)

Supplementary Table 1. Demographic information for Cohorts 1, 2 (sequences of human antibodies post COVID-19 vaccination), and 3 (human sera samples used in competition ELISA).

MAb ID (Cohort 1)	Participant ID (Cohort 1)	MAb ID (CoV-AbDab)	Exposure (CoV-AbDab)	PMID (CoV-AbDab)
M30	P3	368.13.C.0116	SARS-CoV-2 infection	35168246
M13	P3	R568-2E7	SARS-CoV-2 infection	34973165
M14	P3	mAb-31	SARS-CoV-1 infection	32540900
		VA014_14	SARS-CoV-2 vaccination	35477023
		368.01a.A.0069	SARS-CoV-2 infection	35168246
		368.02a.C.0003	SARS-CoV-2 infection	35168246
		368.07.C.0027	SARS-CoV-2 infection	35168246
		368.10.D.0009	SARS-CoV-2 infection	35168246
		368.10.D.0257	SARS-CoV-2 infection	35168246
		368.20.B.0010	SARS-CoV-2 infection	35168246
		368.20.B.0033	SARS-CoV-2 infection	35168246
M15	P3	368.20.B.0034	SARS-CoV-2 infection	35168246
		368.22.A.0007	SARS-CoV-2 infection	35168246
		H712427+K711927	SARS-CoV-1 infection	33442694
		H712443+K711941	SARS-CoV-2 infection	33442694
		PDI-38	SARS-CoV-2 infection	34610292
		R121-3G10	SARS-CoV-2 infection	34973165
		R259-1F4	SARS-CoV-2 infection	34973165
		Shiakolas_53181-5	SARS-CoV-2 infection	35241839
		XG001	SARS-CoV-2 infection	33485405

Supplementary Table 2. Antibodies from CoV-AbDab matching Cohort 1 antibody sequences. Monoclonal antibody sequences from Cohort 1 that matched entries in CoV-AbDab are listed based on identical V and J gene usage and >70% CDR3 similarity in both heavy and light chains. The table includes the type of SARS-CoV-2 exposure (infection or vaccination) of the matched antibodies as reported in CoV-AbDab, along with references (PMIDs) to the original studies from which these antibodies were derived and annotated.

Study	Antigen	Number of sequences	Number of individuals	PMID
Dugan *et al.,* 2020	Influenza	255	25	33298562
Andrews *et al*., 2015	Influenza	170	21	26631631
Raymond *et al*., 2015	Influenza	1	1	29255041
Ekiert *et al*., 2012	Influenza	1	1	22982990
Zost *et al*., 2019	Influenza	33	13	31875553
McCarthy *et al*., 2018	Influenza	12	1	29343437
Guthmiller *et al*., 2021a	Influenza	37	10	34078743
Watanabe *et al*., 2019	Influenza	12	4	31100267
Whittle *et al*., 2014	Influenza	65	1	24501410
Dunand *et al*., 2016	Influenza	12	4	27281570
Wrammert *et al*., 2011	Influenza	45	4	21220454
Joyce *et al*., 2016	Influenza	457	6	27453470
Corti *et al*., 2011	Influenza	9	N/A	21798894
Kallewaard *et al*., 2016	Influenza	20	1	27453466
Guthmiller *et al*., 2021b	Influenza	1948	20	34942633
Henry *et al*., 2019a	Influenza	197	31	30795982
Stadlbauer *et al*., 2019	Influenza	3	1	31649200
Grandea *et al*., 2010	Influenza	17	N/A	20615945
Turner *et al*., 2020	Influenza	300	3	32866963
Henry *et al*., 2019b	Influenza	80	11	31434733
Li *et al*., 2012	Influenza	21	5	22615367
Andrews *et al*., 2017	Influenza	29	13	28783708
McCarthy *et al*., 2019	Influenza	8	1	31843892
Kanekiyo *et al*., 2019	Influenza	2	1	30742080
Coelho *et al*., 2020	Malaria	61	5	33048842
Coelho *et al*., 2024	Malaria	132	8	39127706

Supplementary Table 3. In-house compiled dataset of monoclonal antibodies (mAbs) generated in response to influenza infection, influenza vaccination, or malaria vaccination. Studies lacking information on the number of individuals have been marked as "N/A.".

**Data collection**	
Grid type	UltrAuFoil 1.2/1.3
Microscope/voltage/detector	Titan Krios/300 kV/Gatan K3 summit
Magnification	81,000
Recording mode	counting
Total dose	57.64 e-/Å^2/s
Pixel size	0.826 Å/pixel
Defocus range	–1 to –2.5 μm
No. micrographs used	5,945
Total particles picked	2,717,456
**Model Validation**	
**Composition (#)**	
Chains	3
Atoms	6336
Residues	Protein: 818
Ligands	NAG: 7
**Bonds (RMSD)**	
Length (Å) (# > 4sigma)	0
Angles (°) (# > 4sigma)	0
MolProbity score	1.27
Clash score	2.31
**Ramachandran plot (%)**	
Outliers	0
Allowed	3.87
Favored	96.13
Rotamer outliers (%)	1.85
Cbeta outliers (%)	0
**Peptide plane (%)**	
Cis proline/general	1
Twisted proline/general	0
CaBLAM outliers (%)	2.5
**ADP (B-factors) min/max/mean**	
Protein	90.1
Ligand	90.1
**Data**	
Lengths (Å)	66.08, 100.77, 149.51
Angles (°)	90.00, 90.00, 90.00
Supplied Resolution (Å)	3.37
**Resolution Estimates (Å)**	

d FSC (half maps; 0.143)	3.37
d 99 (full/half1/half2)	2.5
d model	2.2
d FSC model (0/0.143/0.5)	1.7/1.9/3.5
Map min/max/mean	-0.00/2.11/0.02
**Model vs. Data**	
CC (mask)	0.81
CC (box)	0.67
CC (peaks)	0.53
CC (volume)	0.82
Mean CC for ligands	0.70

Supplementary Table 4: Cryo-EM data collection and model validation statistics

FIGURES

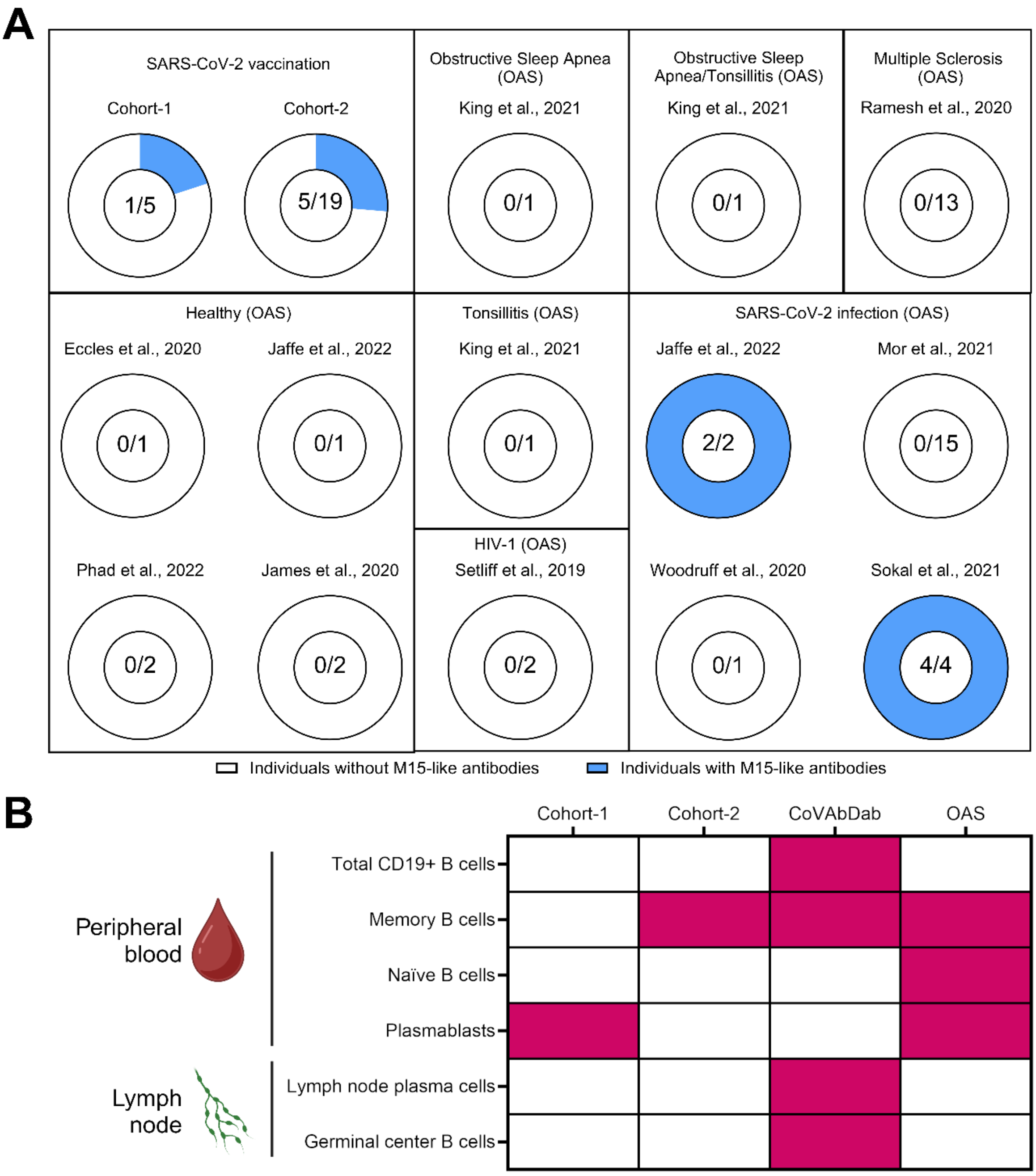

Supplementary Figure 1. Identification of human B cells harboring M15-like antibody sequences.
(A) Frequency of individuals with M15-like antibodies from Cohort 1, Cohort 2, and OAS database, represented as donut plots.
(B) The presence of the B cell subtype (y-axis) expressing an M15-like BCR/antibody in the corresponding cohort or database where the sequence was identified is shown in pink. Due to variability in sequencing depth across studies, a quantitative analysis comparing frequencies across anatomical sites was not feasible.

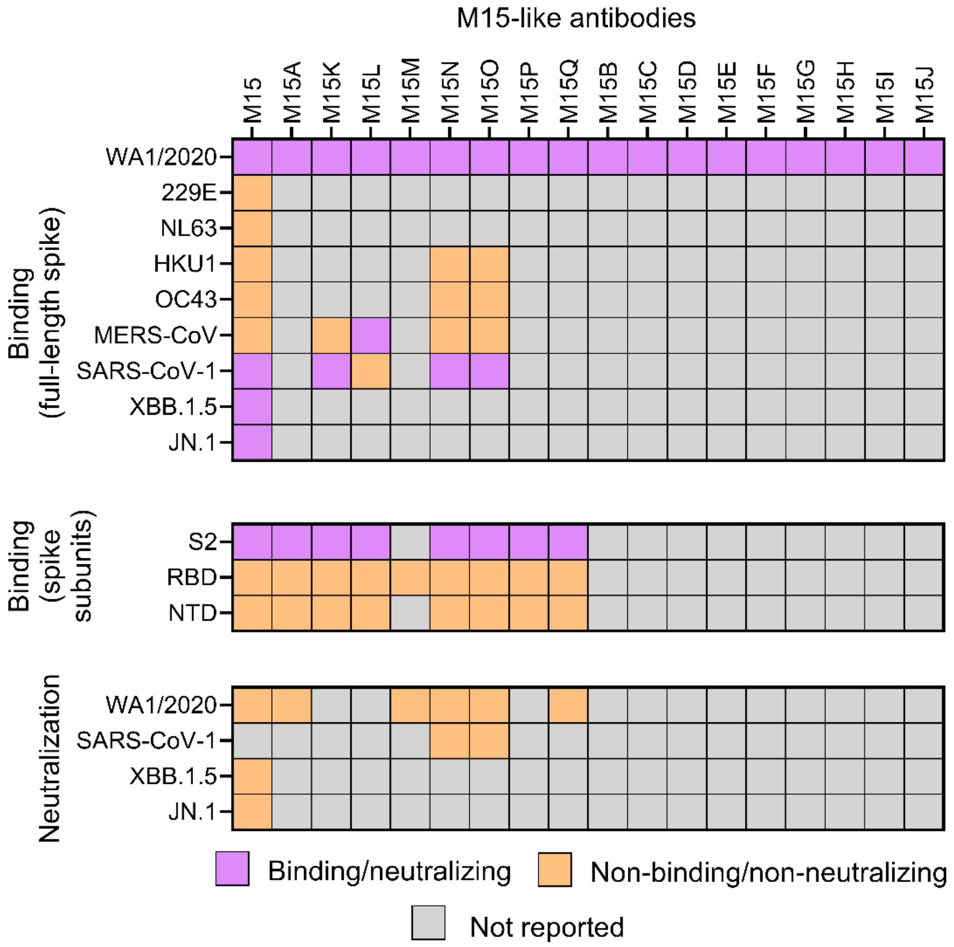

Supplementary Figure 2. Binding and neutralization activity of M15-like antibodies from CoVAbDab.

Binding and neutralization activity of M15-like antibodies, as reported in the individual studies in CoV-AbDab. These data confirm that M15-like antibodies are S2-binding non-neutralizing molecules.

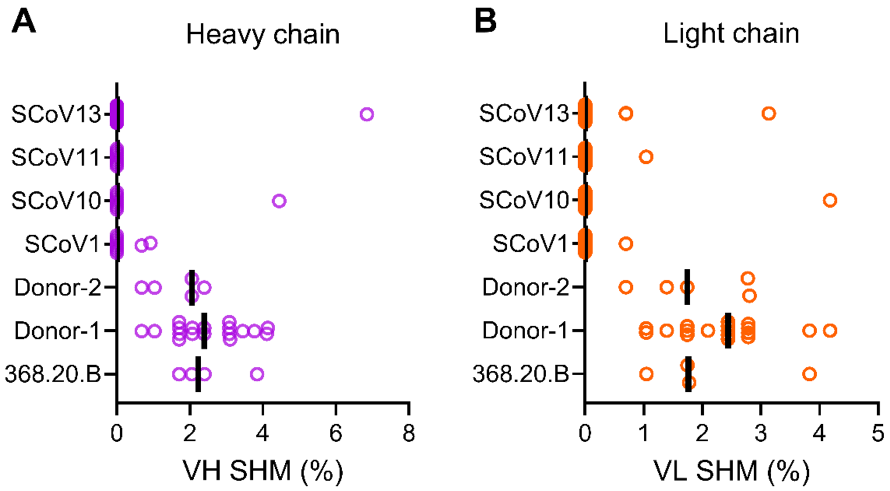

Supplementary Figure 3. Extent of SHM in sequences from M15-like clonotypes.
SHM rates in the **(A)** heavy and **(B)** light chain variable regions among the seven M15-like clonal lineages constructed from individuals with at least three M15-like sequences. The bar represents the median of SHM values.

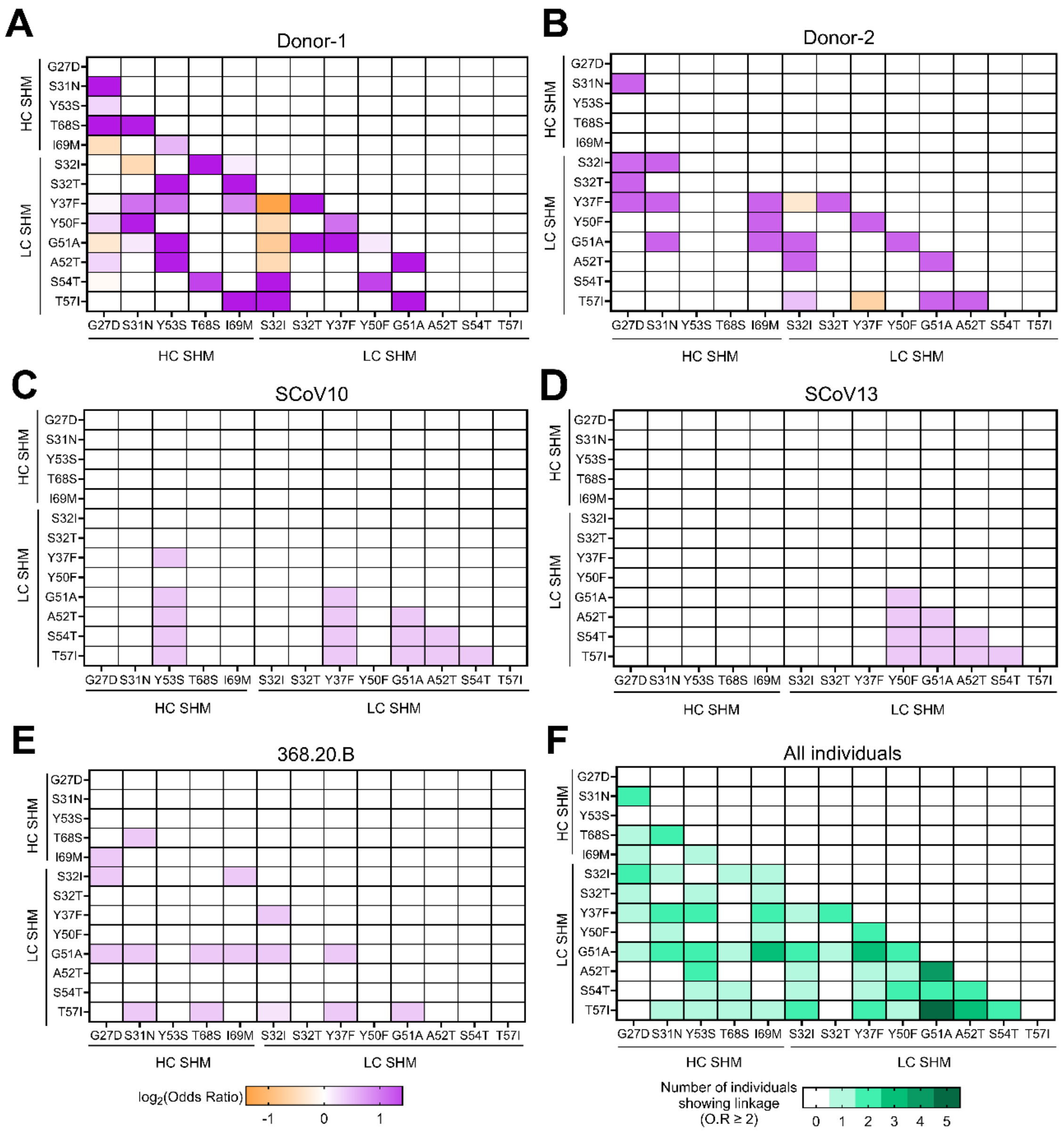

Supplementary Figure 4. Linkage between convergent SHM in M15-like clonotypes.
(A-E) Heatmaps representing linkage between each pair of heavy and light chain convergent mutations within each M15-like clonotype. Linkage is represented as $\log_2$ (Odds Ratio).
(F) Heatmap representing the number of individuals showing significant linkage (defined as odds ratio (O.R) ≥2) between each pair of convergent mutations in heavy and light chains. The location of mutations (heavy/light chain) is mentioned next to or below the axes. Since the heatmap is symmetric, values in the upper right triangle are not shown. Individuals SCoV1 and SCoV11 were omitted due to lack of linkage between any pair of mutations.

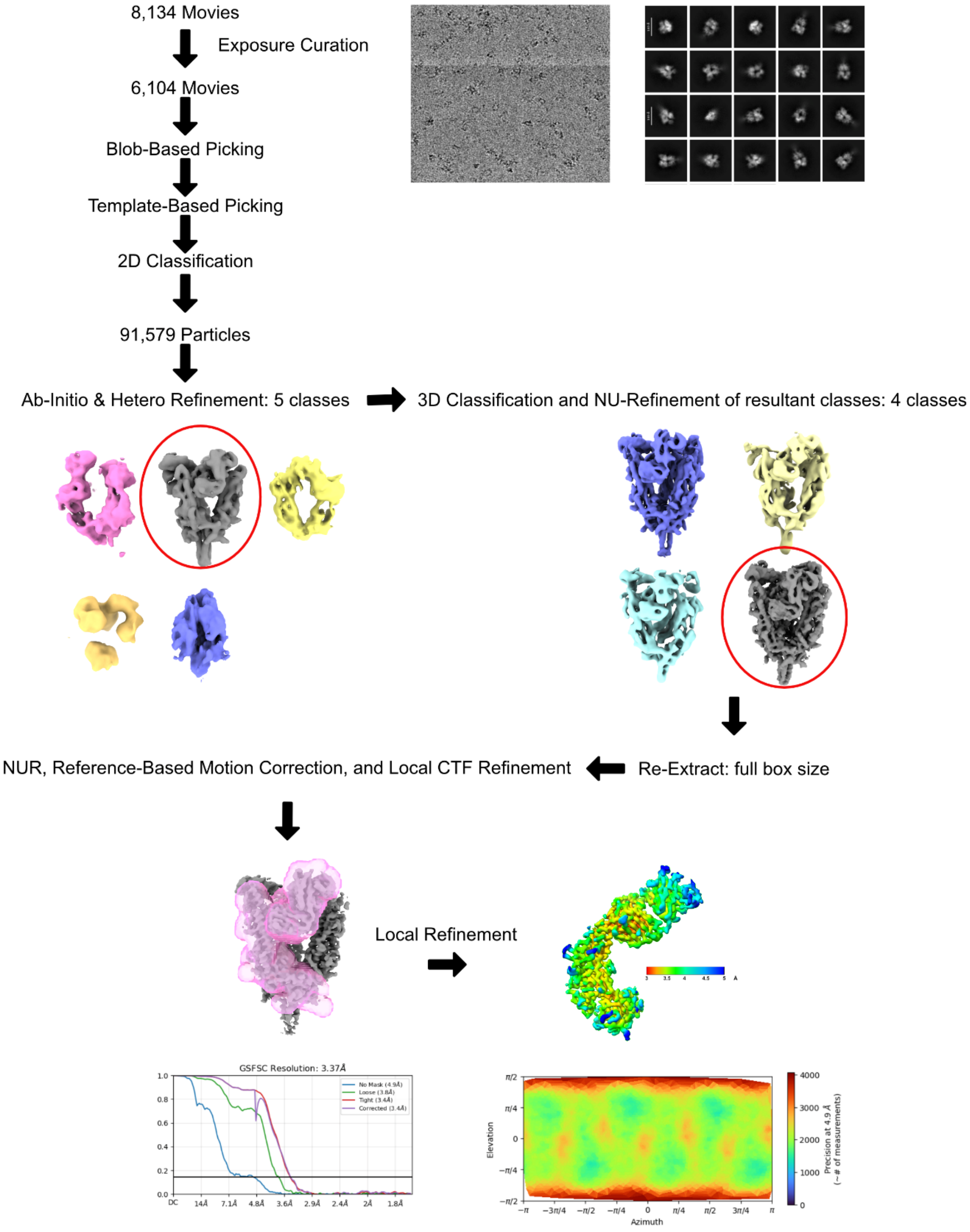

Supplementary Figure 5: CryoEM data processing scheme.

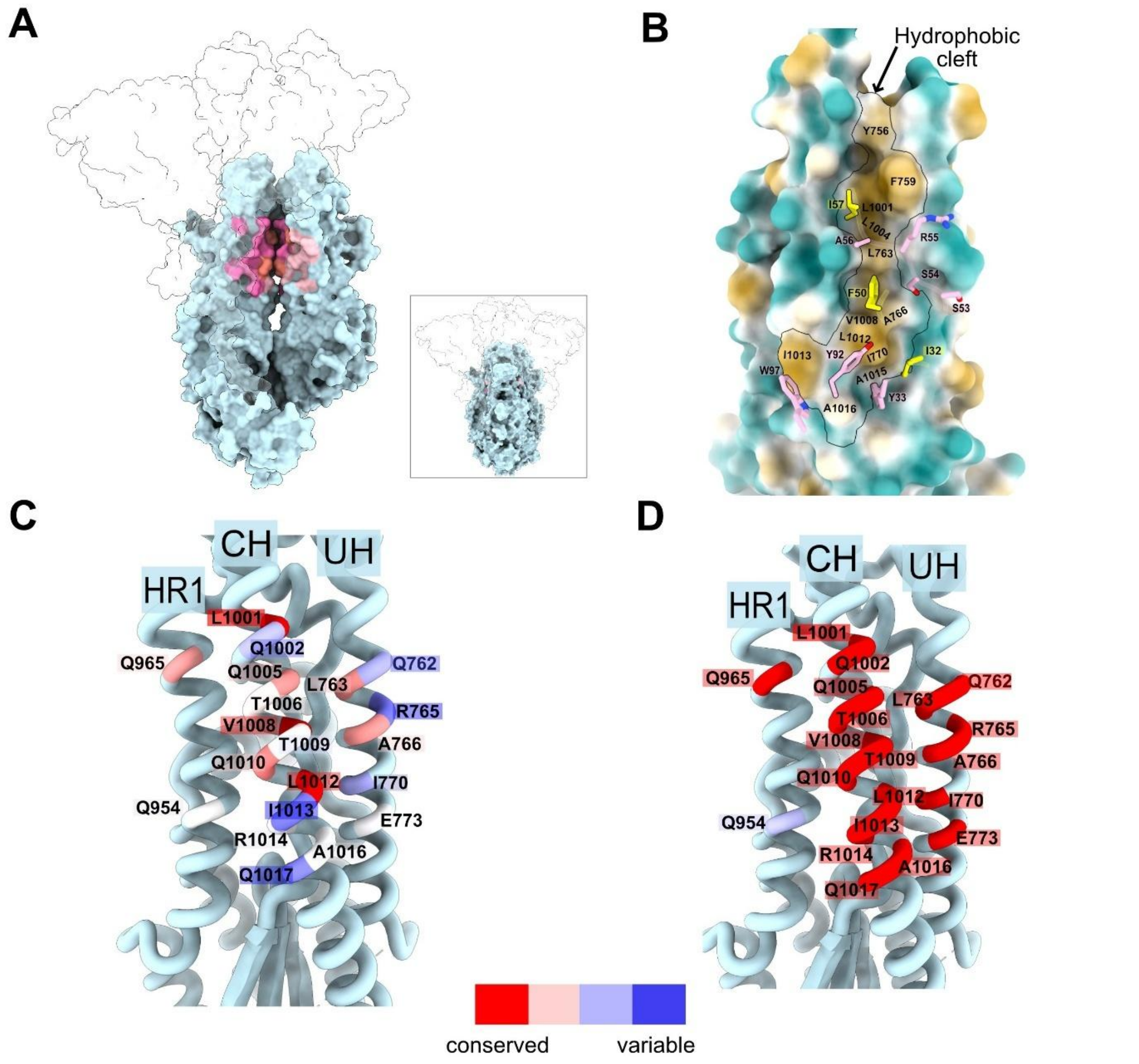

Supplementary Figure 6. Physicochemical properties and conservation of the central interface epitope

(A) Position of the M15 epitope at the interprotomer interface of the full-length SARS-CoV2 spike in the canonical closed conformation (PDB 6ZP2). S1 domains and one out of three S2 protomers are rendered in a transparent white to permit visualization of the occluded M15 binding footprint. S2 residues are colored, as in figure 4C (heavy chain contacts in magenta, light chain contacts in pink). S2 Residues contacting both heavy at light chains are rendered in salmon.

(B) Light chain residues of M15 interact with the hydrophobic cleft in S2 (outlined in black). S2 surface is colored according to the hydrophobicity (Kyte-Doolittle hydrophobicity score) of S2 residues. Hydrophobicity values are depicted in a color palette ranging from camel hair (hydrophobic) to robin's egg blue (hydrophilic). M15 light chain contacts are displayed in pink, with convergent and clonotype-enriched mutations highlighted in yellow.

(C) Conservation of M15 contacts across hCoVs 229E, NL63, HKU1, HKU4, HKU5, OC43, MERS-CoV, SARS-CoV and SARS-CoV-2, displayed on ribbon structure of S2.

(D) Conservation of M15 contacts across sarbecoviruses SARS-CoV-1, SARS-CoV-2 WA1/2020, and SARS-CoV-2 variants B.1.1.7, P.1, B.1.617.2, BA.1, BA.2.86, BA.5, BQ.1.1, XBB.1.5, and JN.1 displayed on the ribbon structure of S2.

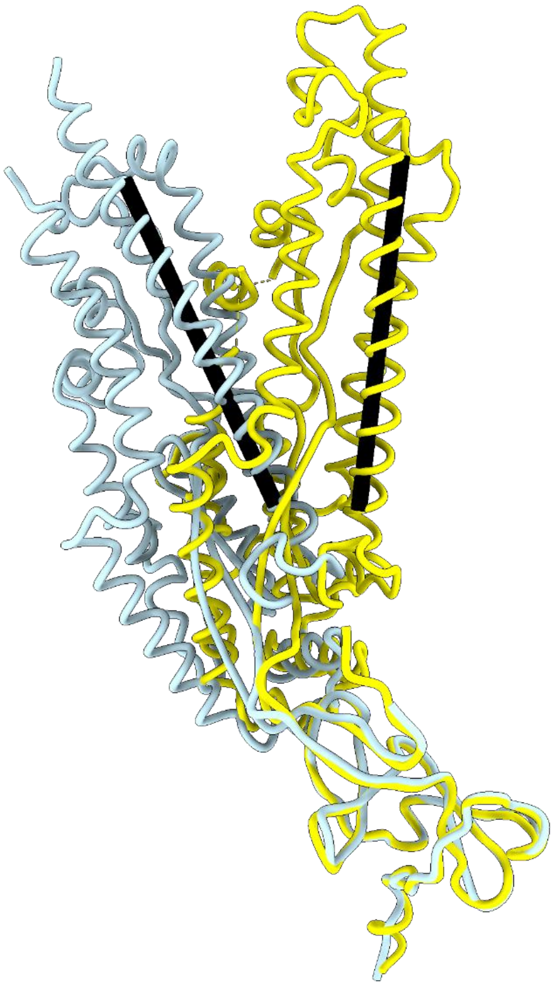

Supplementary Figure 7. The M15-bound S2 protomer (gray) splays outwards from an axis defined by the central helix.
S2 protomer structures derived from the M15-S2 complex (gray) and the structure of S2 in the absence of M15 (yellow; PDB 8VQB) were superposed at residues 1010-1040. Axes were defined by the central helix between residues 998-1028 in each S2 model. The angle subtending said axes measures 32°.

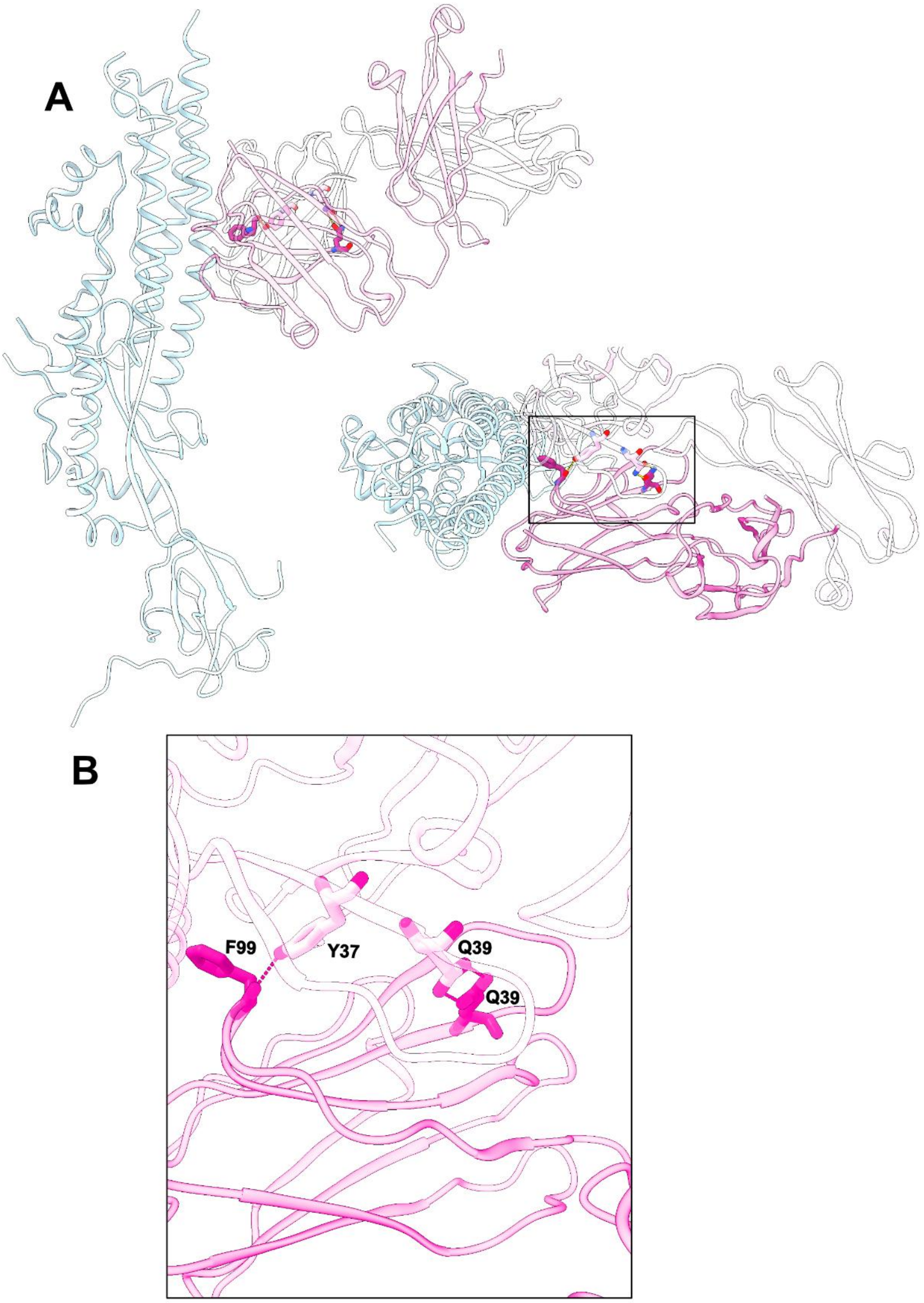

Supplementary Figure 8. Inter-chain hydrogen bonds between residues within M15 Fab heavy and light chain variable regions.
(A) Inter-chain contacts are displayed in stick representation, situated within the ribbon structure of M15-S2 protomer structure; lateral view, left; top-down view, right.
(B) Close-up view of inter-Fab contacts. Heavy and light chain residues are colored in dark and light pink, respectively.

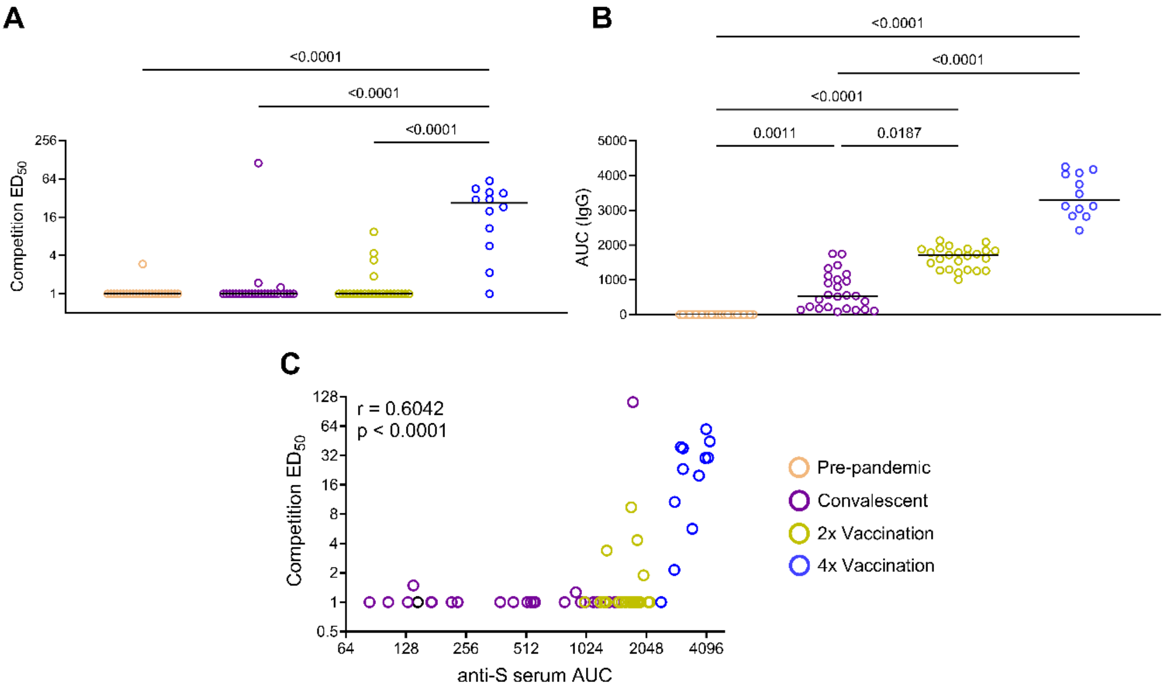

Supplementary Figure 9. Competition ELISA between serum antibodies and M15 mAb.

(A) Serum competition ELISA (unnormalized) between biotinylated M15 and human sera from pre-pandemic, SARS-CoV-2 convalescent, two doses of SARS-CoV-2 vaccination and four doses of SARS-CoV-2 vaccination. Competition is represented as unnormalized ED_{50} values. ED_{50} values less than 1 were set to a constant value of 1.

(B) Anti-spike serum ELISA titers from pre-pandemic, SARS-CoV-2 convalescent, two doses of SARS-CoV-2 vaccination, and four doses of SARS-CoV-2 vaccination sera samples. Serum titers are computed as area under the curve (AUC) values.

(C) Correlation between competition ED_{50} values and anti-spike serum titers computed as AUC values of all samples. The circles are colored based on exposure groups. Comparisons between exposure groups in (A) and (B) were performed using the Kruskal-Wallis test followed by Dunn's multiple correction. Spearman correlation was performed to estimate the r-coefficient and *p*-values for the correlation analysis in (C). The p-values represent total correlation across infection, two-dose vaccination, and four-dose vaccination groups.
